# Structural insights into human brain–gut peptide cholecystokinin receptors

**DOI:** 10.1038/s41421-022-00420-3

**Published:** 2022-06-07

**Authors:** Yu Ding, Huibing Zhang, Yu-Ying Liao, Li-Nan Chen, Su-Yu Ji, Jiao Qin, Chunyou Mao, Dan-Dan Shen, Lin Lin, Hao Wang, Yan Zhang, Xiao-Ming Li

**Affiliations:** 1 Department of Neurobiology and Department of Neurology of Second Affiliated Hospital, Zhejiang University School of Medicine, Hangzhou, Zhejiang China; 2 NHC and CAMS Key Laboratory of Medical Neurobiology, MOE Frontier Center of Brain Science and Brain-machine Integration, School of Brain Science and Brain Medicine, Zhejiang University, Hangzhou, Zhejiang China; 3 Department of Biophysics and Department of Pathology of Sir Run Run Shaw Hospital, Zhejiang University School of Medicine, Hangzhou, Zhejiang China; 4 Liangzhu Laboratory, Zhejiang University Medical Center, Hangzhou, Zhejiang China; 5 Zheijang Provincial Key Laboratory of Immunity and Inflammatory Diseases, Hangzhou, Zhejiang China; 6 Center for Brain Science and Brain-Inspired Intelligence, Research Units for Emotion and Emotion Disorders, Chinese Academy of Medical Sciences, China/Guangdong-Hong Kong-Macao Greater Bay Area, Joint Institute for Genetics and Genome Medicine between Zhejiang University and University of Toronto, Hangzhou, Zhejiang China

**Keywords:** Cryoelectron microscopy, Cell biology

## Abstract

The intestinal hormone and neuromodulator cholecystokinin (CCK) receptors CCK1R and CCK2R act as a signaling hub in brain–gut axis, mediating digestion, emotion, and memory regulation. CCK receptors exhibit distinct preferences for ligands in different posttranslational modification (PTM) states. CCK1R couples to G_s_ and G_q_, whereas CCK2R primarily couples to G_q_. Here we report the cryo-electron microscopy (cryo-EM) structures of CCK1R–G_s_ signaling complexes liganded either by sulfated cholecystokinin octapeptide (CCK-8) or a CCK1R-selective small-molecule SR146131, and CCK2R–G_q_ complexes stabilized by either sulfated CCK-8 or a CCK2R-selective ligand gastrin-17. Our structures reveal a location-conserved yet charge-distinct pocket discriminating the effects of ligand PTM states on receptor subtype preference, the unique pocket topology underlying selectivity of SR146131 and gastrin-17, the conformational changes in receptor activation, and key residues contributing to G protein subtype specificity, providing multiple structural templates for drug design targeting the brain–gut axis.

## Introduction

Cholecystokinin (CCK) receptor family belongs to class-A sevenfold transmembrane G protein-coupled receptors, and is divided into CCK1 receptor (CCK1R) and CCK2 receptor (CCK2R)^1^. CCK1R is mainly distributed in the gastrointestinal tract^2–4^, peripheral nervous system^5,6^, and some regions of the brain, e.g., the area postrema, the nucleus tractus solitarius, and the hypothalamus^7–10^. Conversely, CCK2R is primarily expressed in the brain, particularly in the cortex and the limbic structures including the amygdala, the hippocampus, and the nucleus accumbens^11–14^ and selected regions in the gastrointestinal tract, including gastric epithelial parietal cells^15^, pancreatic acinar cells^16^, myenteric neurons^17^, and human peripheral blood mononuclear cells^18^. Hence, CCK receptors (CCKRs) regulate a variety of physiological functions including digestion, satiety, emotion regulation, pain sensation, and memory process^18–25^. Besides, CCKRs are expressed in the brain–gut axis which is a region critical for the transmission of information between gut and brain including satiation signals. For these functions, there are already some specific drugs designed for CCK1R, such as ceruletide, or for CCK2R, including proglumide and pentagastrin. Although all of them have been used for gastrointestinal diseases, they and some drugs under clinical trial also have the potential to treat CNS diseases, including pain and anxiety^26,27^. Meanwhile, various CCKR-targeted drugs have been developed, yet most of them were ultimately removed from the market due to questionable efficacy, lack of target specificity, or severe adverse effects^28,29^. This highlights the need for investigation of the structure of CCK1R and CCK2R to understand agonist binding, receptor activation and thus promote structure-based drug discovery.

**Fig. 1:**
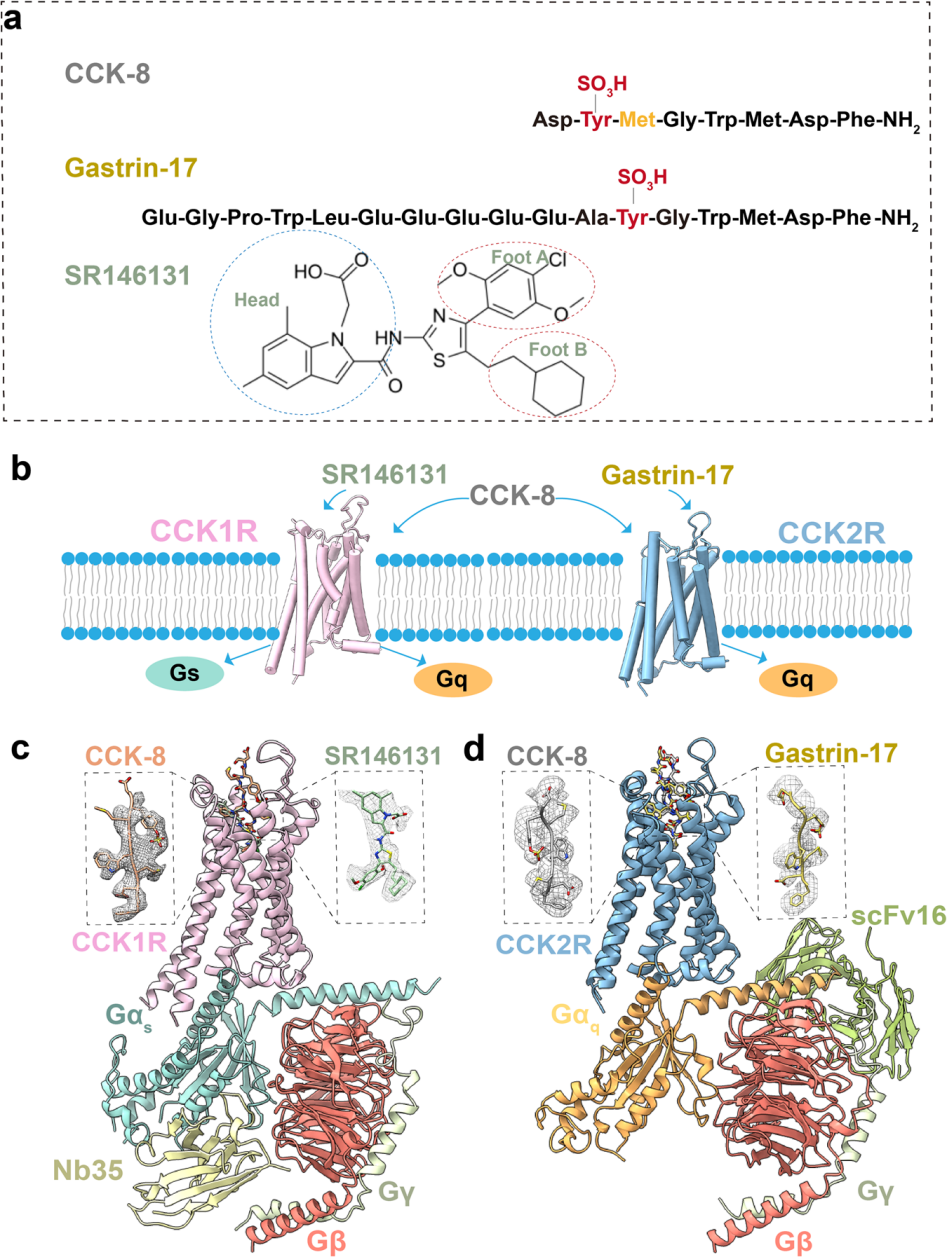
Overall structures of CCK1R and CCK2R signaling complexes. **a** Molecular formula of CCK-8, gastrin-17, and SR146131. Sulfated tyrosines are colored red and the amino acid differs between gastrin-17 and CCK-8 is colored orange. **b** Schematic diagram showing various ligands signaling through CCK1R and CCK2R. **c**, **d** Representation of CCK-8-bound CCK1R–G_s_ complex and SR146131-bound CCK1R–G_s_ complex. CCK1R, pink; CCK-8, sienna; SR146131, dark sea-green; Gα_s_, cyan; Gβ, salmon; Gγ, beige; Nb35, light yellow (**c**). Representation of CCK-8-bound CCK2R–G_q_ complex and gastrin-17-bound CCK2R–G_q_ complex. CCK2R, blue; CCK-8, gray; gastrin-17, yellow; Gα_q_, orange; Gβ, salmon; Gγ, beige; scFv16, lawn green (**d**).

The endogenous ligands of CCKRs are CCK and gastrin. They exist in multiple molecular forms, sharing the same evolutionarily conserved pentapeptide motif, which comprises the minimal sequence required for biological activity and receptor activation (Fig. 1a). CCK-8 predominates in the brain, and gastrin-17 accounts for 90% of gastrin in the human body, inducing acid release^30^. Notably, posttranslational modifications (PTMs) are critical for CCK and gastrin activity, including sulfation of the tyrosine (TYS) at position 7 from the C-terminus in CCK and at position 6 in gastrin (Fig. 1a). Intriguingly, CCK2R binds sulfated and non-sulfated ligands equally well, whereas CCK1R exclusively responds to sulfated CCK. In addition, CCK-8 stimulates both CCKRs and gastrin-17 is highly selective for CCK2R (Supplementary Fig. S1a)^31,32^. Apart from ligand selectivity, huge differences in downstream G protein signaling are present between CCK1R and CCK2R. CCK1R couples to the stimulatory G protein G_s_ and G_q_ (Fig. 1b; Supplementary Fig. S1b, c), and induces diverse effects under different pathophysiological conditions^33^. For example, CCK1R-activated G_q_ signaling is required for insulin secretion under low-glucose conditions, whereas G_s_ signaling contributes more strongly to insulin secretion under high-glucose conditions^34^. It is noteworthy that CCK2R exclusively couples to G_q_ (Supplementary Fig. S1b, c) and may contribute to the generation of anxiety, fear, and the increase of pancreas growth as well as the formation of preneoplastic lesions^35–40^, potentially indicating a different CCKR activation mechanism. Owing to the paucity of knowledge about the molecular mechanism of PTMs’ effects on ligand selectivity, receptor activation, and G protein specificity, additional studies to delineate the molecular mechanism underlying CCKR activation are urgently needed.

In the present study, we determined the cryo-electron microscopy (cryo-EM) structures of CCK1R in complex with G_s_, stimulated by sulfated CCK-8 (hereafter referred to as CCK-8 unless otherwise indicated) and a CCK1R-selective small-molecule agonist SR146131^41^, respectively. For comparison, we also resolved the cryo-EM structures of CCK2R–G_q_ bound to CCK-8 and sulfated gastrin-17 (hereafter referred to as gastrin-17 unless otherwise indicated), respectively. These structures provide unprecedented structural insights into the agonist selectivity, G protein selectivity, and receptor activation mechanism of CCK1R and CCK2R. The structures also provide multiple templates for the rational design of novel therapeutics against not only metabolic diseases such as obesity and metabolic syndrome but also neuropsychiatric disorders such as anxiety and pain.

## Results

### Overall structures of CCK1R and CCK2R signaling complexes

For structural studies with different transducer-coupled CCKRs (CCK1R–G_s_ and CCK2R–G_q_), human CCK1R and CCK2R with 15-residue depletion at the C-terminus were subcloned into pFastBac1 vector with an N-terminal Flag-tag, along with a LgBiT and double maltose-binding protein (MBP) affinity tag at the C-terminus to facilitate the protein expression and purification^42^. CCK1R was co-expressed with human G_s_ protein in Sf9 insect cells to form the CCK1R–G_s_ complexes and activated by CCK-8 and by CCK1R-selective agonist SR146131, respectively. To obtain the human CCK2R–G_q_ complex bound to CCK-8 or the CCK2R-selective agonists gastrin-17, we co-expressed human CCK2R in Sf9 insect cells with human wild-type G_q_ heterotrimer, except that the αN helix of Gα_q_ was replaced with that of Gα_i_ to enable the binding of a single-chain variable fragment (scFv16)^43^. All complexes were purified to homogeneity for single-particle cryo-EM analysis (Supplementary Fig. S2).

Structures of the CCK-8–CCK1R–G_s_, SR146131–CCK1R–G_s_, CCK-8–CCK2R–G_q_, and gastrin-17–CCK2R–G_q_ complexes were determined with global resolutions of 3.2, 3.0, 3.1, and 3.1 Å, respectively (Fig. 1c, d; Supplementary Figs. S2, S3 and Table S1). The relatively high-resolution density maps of the four complexes allowed confident modeling of the CCK-8 and SR146131 molecules, the N-terminal 8 residues of gastrin-17, most portions of the two receptors, and their corresponding G proteins. Notably, although both CCKRs adopt the canonical seven-transmembrane architecture, they differ from other reported class-A GPCRs with a shortened TM4 helix, which is attributed to the unwinding of the helix at the extracellular half caused by the presence of P^4.59^ and the additional P^4.61^ (the superscripts refer to the Ballesteros–Weinstein number)^44^.

### Endogenous ligand recognition of CCKRs

**Fig. 2:**
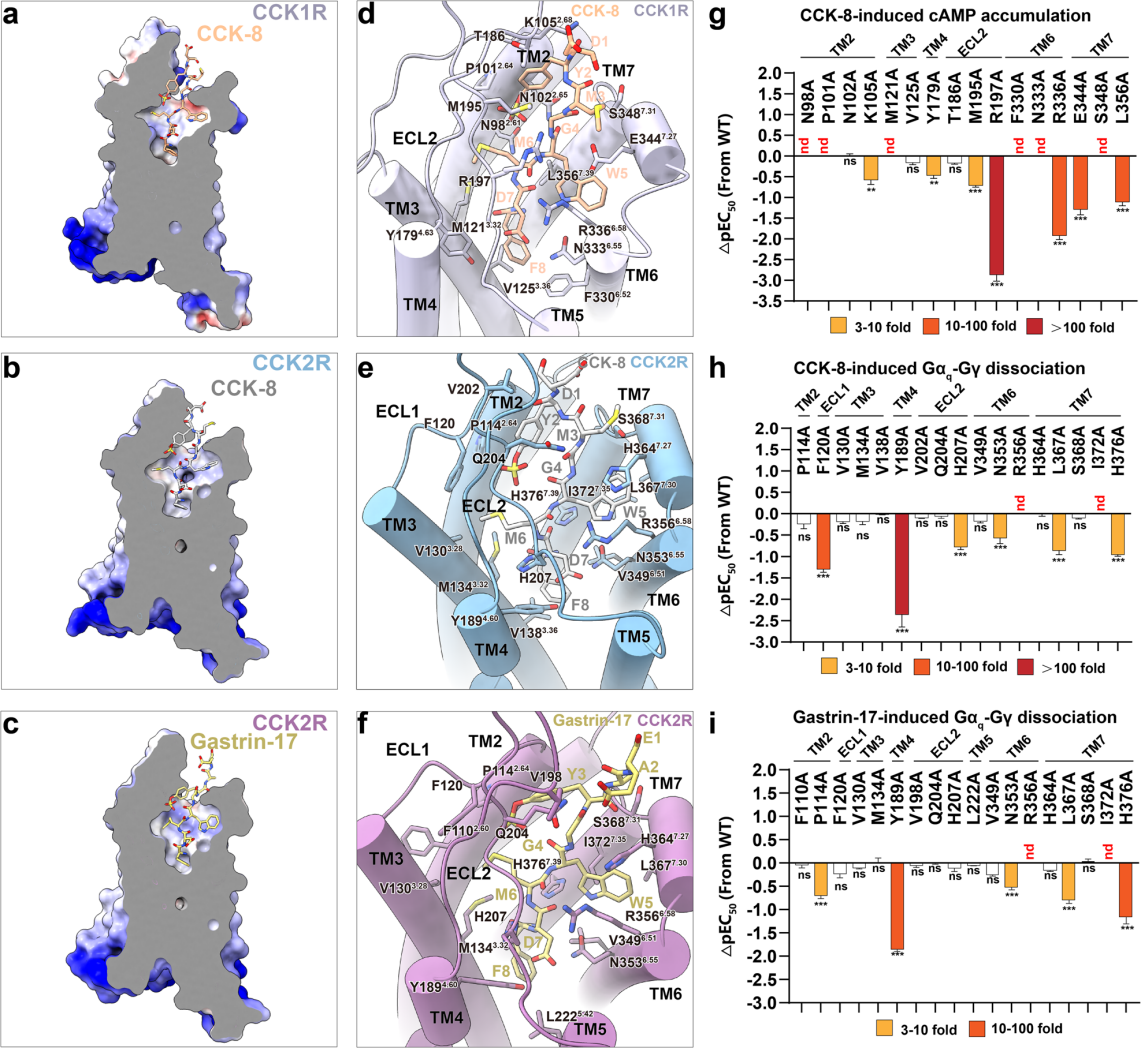
Endogenous ligand recognition of CCK1R and CCK2R. **a–c** Electrostatic potential map of CCK-8-binding pocket in CCK1R (**a**), CCK2R (**b**), and gastrin-binding pocket in CCK2R (**c**). **d–f** Detailed interactions between CCK-8 (sienna) and CCK1R (light slate blue) (**d**); between CCK-8 (gray) and CCK2R (blue) (**e**); between gastrin-17 (yellow) and CCK2R (purple) (**f**). **g** Mutagenesis analysis of residues involved in CCK-8 binding using CCK-8-induced cAMP accumulation assay. **h** Mutagenesis analysis of residues involved in CCK-8 binding using CCK-8-induced Gα_q_–Gγ dissociation assay. **i** Mutagenesis analysis of residues involved in gastrin-17 binding using gastrin-17-induced Gα_q_–Gγ dissociation assay. Bars represent differences in calculated potency (pEC_50_) for representative mutants relative to the wild-type receptor (WT). Data were colored according to the extent of effect (orange, 3–10-fold reduction of EC_50_; tomato, 10–100-fold reduction of EC_50_; red, > 100-fold reduction of EC_50_). ns, no significance; **P < 0.001, ***P < 0.0001 (one-way ANOVA followed by Dunnett’s post hoc test, compared with response of WT). nd, pEC_50_ change went beyond the detection range.

It remains mysterious that the CCKRs with highly conserved orthosteric binding pocket display distinct affinity and potency on various endogenous ligands, according to previous homology modeling and mutagenesis studies^45^. The newly determined cryo-EM structures in our work reveal that CCK and gastrin indeed adopt similar linear configurations perpendicular to the bilayer, penetrate into the receptor transmembrane domains (TMDs), and engage CCKR through extensive electrostatic and hydrophobic interactions with residues from all the extracellular loops (ECLs) and TM helices except TM5 (Fig. 2a–f). Although CCK-8 is located in a similar orthosteric pocket in CCK1R and CCK2R, the binding modes significantly differ (Fig. 2d, e). TYS of CCK-8 inserts into the hydrophobic pocket formed by P114^2.64^, F120^ECL1^, and Q204^ECL2^ in CCK2R (CCK2R-TYS-pocket), while inserting into the pocket formed by N98^2.61^, P101^2.64^, N102^2.65^, K105^2.68^, T186^ECL2^, R197^ECL2^, and M195^ECL2^ in CCK1R (CCK1R-TYS-pocket). N98^2.61^ and R197^ECL2^ form hydrogen bonds with the sulfonic acid group of TYS, vital for CCK-8 binding to CCK1R. The importance of the hydrogen bonds was reflected in our mutagenesis studies, wherein R197^ECL2^A decreased the potency of CCK-8 by over 100-fold and N98^2.61^A abolished the efficacy (Fig. 2g, h; Supplementary Tables S2, S3).

In the CCK2R structures, the C-terminal pentapeptides of gastrin-17 and CCK-8 share almost identical binding poses (Supplementary Fig. S4a). When moving towards the N-terminus, we find that residues TYS–Ala–Glu of a gastrin-17 shift to ECL3 compared to CCK-8. Though gastrin-17 possesses a distinct amino acid sequence and lacks the methionine after TYS, surprisingly, the TYS residue of gastrin-17 inserts into the same hydrophobic pocket of CCK2R as that of CCK-8, except that gastrin-17 interacts more extensively with CCK2R, including F110^2.60^ P114^2.64^, F120^ECL1^, V198^ECL2^, Q204^ECL2^, and S368^7.31^ (Fig. 2f, i; Supplementary Table S4). These differences are most likely related to the missing methionine in gastrin-17, which enables TYS of gastrin-17 to insert closer to ECL2 of CCK2R compared to that of CCK-8. When aligning the CCK-8–CCK1R–G_s_ and gastrin-17–CCK2R–G_q_ complexes, gastrin-17, especially the TYS group formed considerable steric hindrance with CCK1R (Supplementary Fig. S4b), perfectly explaining the critical physiological fact that gastrin-17 selectivity activates CCK2R but not CCK1R in the gut where both CCKRs spontaneously exist and modulate distinctive physiological and pathological processes.

**Fig. 3:**
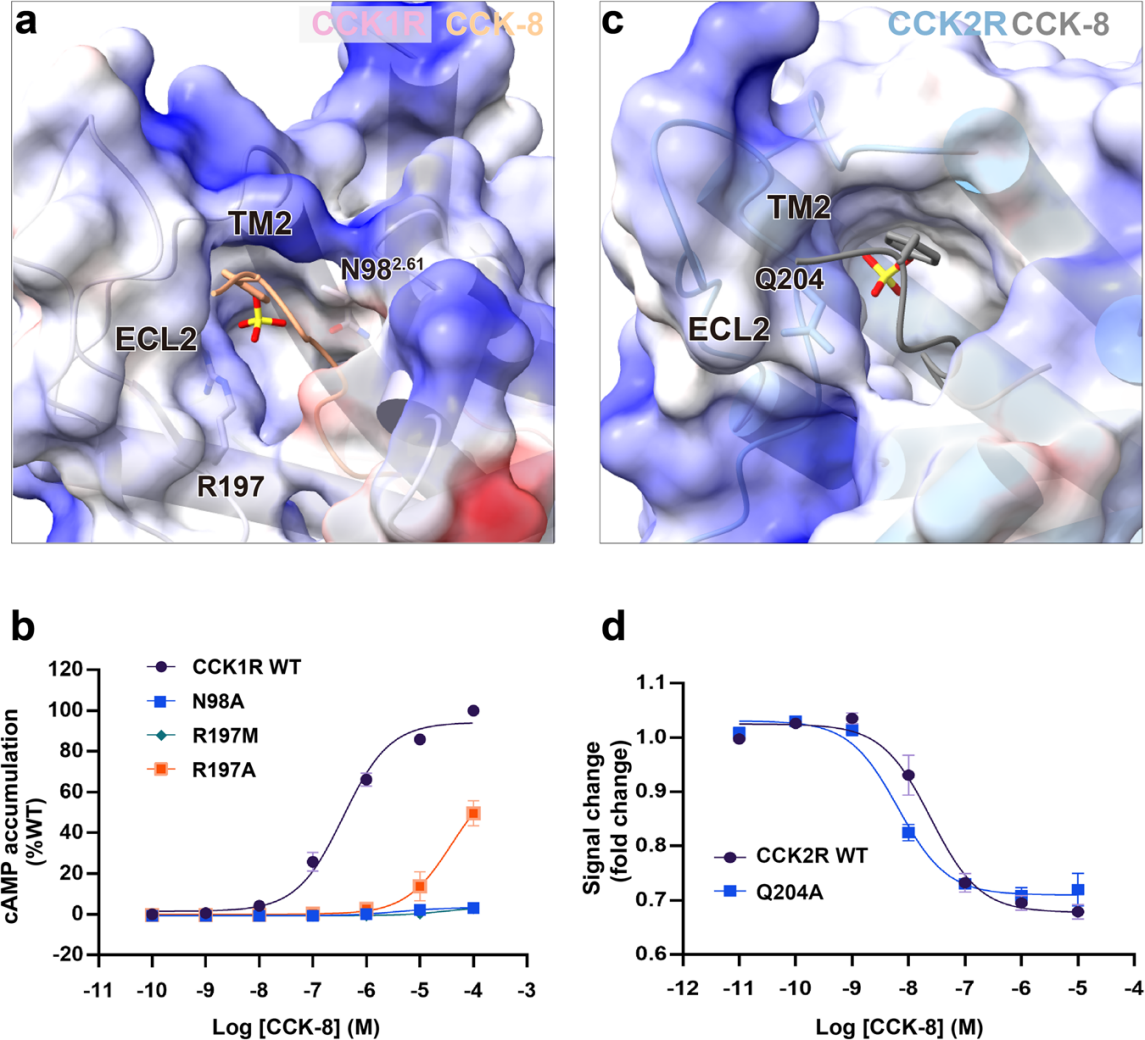
Ligand selectivity of CCK1R for sulfated CCK. **a** Electrostatic potential map of the binding pocket in CCK1R for the sulfated tyrosine of CCK-8. **b** The effect of N98A, R197A, or R197M mutation in CCK1R on CCK-8-induced cAMP accumulation. **c** Electrostatic potential map of the binding pocket in CCK2R for the sulfated tyrosine of CCK-8. **d** The effect of Q204A mutation in CCK2R on CCK-8-induced Gα_q_–Gγ dissociation.

It is worth noting that CCK1R binds and responds to sulfated CCK with a 500- to 1000-fold higher potency than non-sulfated CCK, whereas CCK2R discriminates poorly between sulfated and non-sulfated peptides^45^. According to the two CCK-8-bound structures, the interfaces between CCK-8 and CCKRs and the overall shape of the orthostatic pockets are conserved, consistent with previous prediction^45^. However, the sub-pocket for the TYS of CCK-8 is more positively charged in CCK1R attributed to N98^2.61^, and R197^ECL2^ contributes to a beneficial charge–charge interaction with TYS of CCK-8 (Fig. 3a). Site-directed mutations R197^ECL2^M/A or N98^2.61^A in CCK1R almost abolished CCK-8-triggered activation, implying the critical role of ECL2 in sulfated endogenous ligand recognition (Fig. 3b). On the contrary, the residue in CCK1R structurally corresponding to R197^ECL2^ of CCK2R is an uncharged residue V206 ^ECL2^, and consequently, the binding pocket in CCK2R for TYS of CCK-8, comprised of P114^2.64^, F120^ECL1^, and Q204^ECL2^, is wider and more accessible to the solvent and displays a hydrophobic characterization (Fig. 3c). The substitution of Q204^ECL2^A, the closest polar residue in CCK2R compared to CCK-8, does not affect CCK-8-induced G_q_ signaling of CCK2R measured by NanoBiT assay (Fig. 3d). Therefore, we attribute the fact that CCK2R recognizes non-sulfated and sulfated CCK-8 equally well to the lack of polar interactions between the endogenous ligands and CCK2R.

### Structural basis underlying CCK1R selectivity of SR146131

**Fig. 4:**
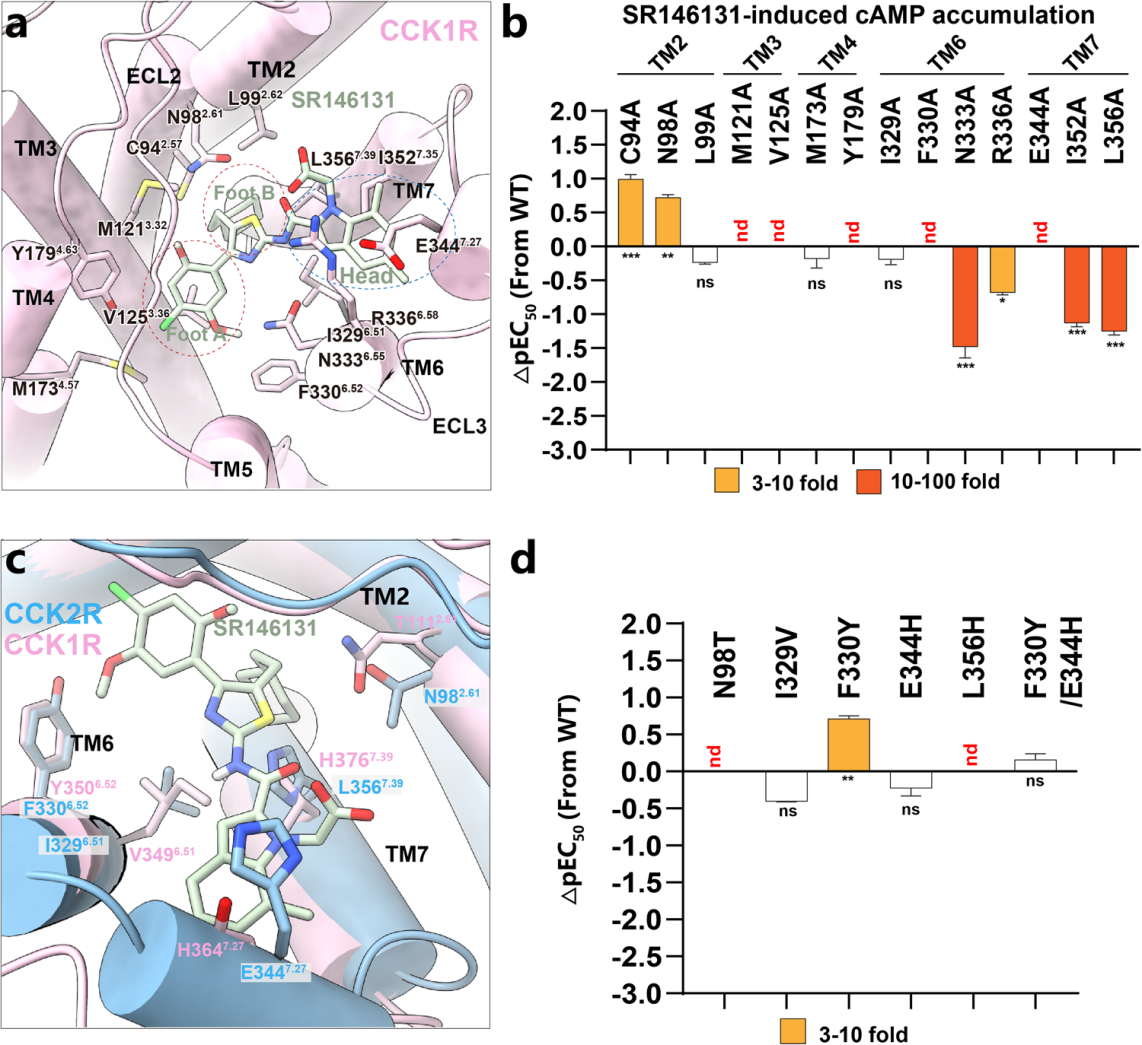
Structural basis underlying ligand selectivity of CCK1R for SR146131. **a** Detailed interactions between SR146131 (dark sea-green) and CCK1R (pink). The structure viewed from the extracellular side shows an interaction network between CCK1R and SR146131. **b** Mutagenesis analysis of residues involved in SR146131 binding using SR146131-induced cAMP accumulation assay. Bars represent differences in calculated SR146131 potency (pEC_50_) for representative mutants relative to WT. Data were colored according to the extent of the effect. ns, no significance, *P < 0.01, **P < 0.001, ***P < 0.0001 (one-way ANOVA followed by Dunnett’s post hoc test, compared with the response of WT). nd, pEC_50_ change went beyond the detection range. **c** Representation of the residues that differ between CCK1R (pink) and CCK2R (blue) in the ligand-binding pocket. **d** Residues involved in ligand recognition in CCK2R are respectively substituted by the corresponding residues in CCK1R, and the effect of each mutation on SR146131-induced cAMP accumulation is shown. Bars represent differences in calculated SR146131 potency (pEC_50_) for representative mutants relative to WT. Data were colored according to the extent of the effect. ns, no significance, **P < 0.001 (one-way ANOVA followed by Dunnett’s post hoc test, compared with the response of WT). nd, pEC_50_ change went beyond the detection range.

SR146131, developed by Sanofi-Synthelabo^41^, is a potent, orally active, and non-peptide agonist that selectively acts on CCK1R with a promising application as atypical antipsychotic drug^46^. In the SR146131–CCK1R structure, TM2–TM4, TM6–TM7, and ECL2–ECL3 of CCK1R participate in the recognition of SR146131 (Fig. 4a). SR146131 occupies a similar position to that of the Phe^1^Asp^2^Trp^4^ motif of CCK-8 (the superscript indicated the residue position from the C-terminus of the peptide) (Supplementary Fig. S4c), which are part of the minimal sequence required for the biological activity of CCK-8 and receptor activation. The head of SR146131 overlies the pocket formed by TM6–ECL3–TM7, and foot A (phenyl ring) stands on TM3–TM6, whereas foot B (cyclohexane ring) stands on TM2 and TM7 (Fig. 4a). L99^2.62^, M121^3.32^, and L356^7.39^ of CCK1R form a hydrophobic pocket for the cyclohexane ring of SR146131. The alanine replacement of L99^2.62^ did not affect the G_s_ signaling, however, M121^3.32^A almost abolished the G_s_ signaling and the L356^7.39^A mutant suffered an over 10-fold potency reduction and 50% decrease in the maximum level of cAMP production. The phenyl ring is stabilized by M173^4.57^, Y179^4.63^, and F330^6.52^. Both Y179^4.63^A and F330^6.52^A substitutions cannot be activated by SR146131 as measured by the cAMP accumulation assay. Substitution of the residues accommodating the head of SR146131 to alanine, including R336^6.58^, E344^7.27^, and I352^7.35^, impaired the CCK1R activation to different extents (Fig. 4b; Supplementary Table S5).

Structure-based sequence alignment revealed several non-conserved residues between CCK1R and CCK2R in the SR146131-binding pocket, including the residues at the positions of 2.61, 6.51, 6.52, 7.27, and 7.39. To understand the molecular basis of the discriminated activity of SR146131 on CCK1R and CCK2R, we then mutated the above non-conserved residues in CCK1R to the corresponding ones in CCK2R and measured SR146131-induced cAMP accumulation. SR146131 activity was completely abolished by either N98^2.61^T or L356^7.39^H mutation, attributed to the steric hindrance by L356^7.39^H and to the loss of polar interactions by N98^2.61^T. In contrast, SR146131-induced receptor activation was not affected by the mutations involving the other two residues (I329^6.51^ and E344^7.27^) (Fig. 4c, d; Supplementary Table S5). In addition, SR146131 also clashes with residue Y^7.43^ in CCK2R, which may also lead to the disability of CCK2R to bind SR146131 (Supplementary Fig. S4c). In conclusion, structures of ligand-bound CCKRs reveal that N98^2.61^, L356^7.39^, and Y360^7.43^ in CCK1R contribute to the subtype selectivity of CCKRs for SR146131.

### Structures of G protein-coupled CCKRs

**Fig. 5:**
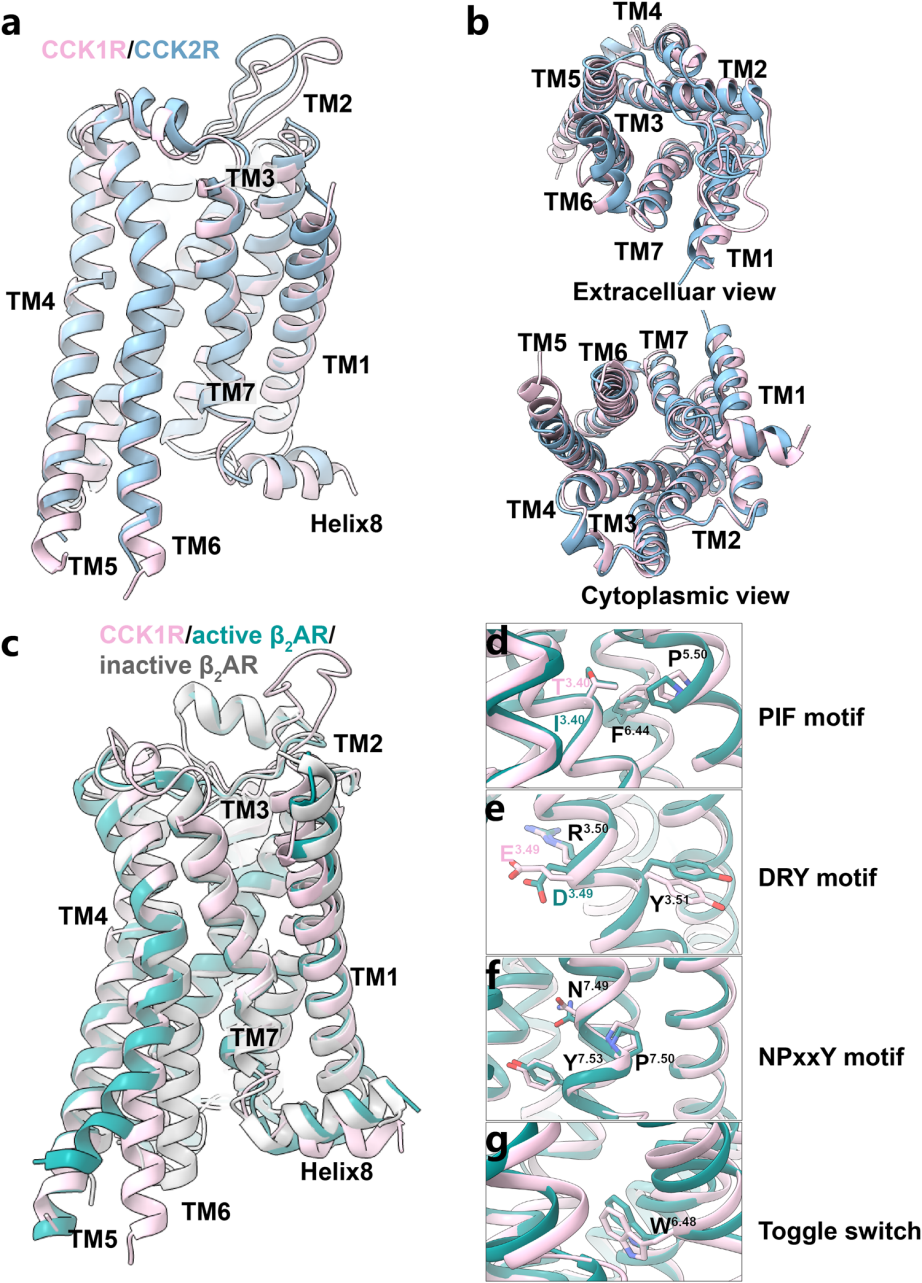
Structural comparisons between CCK1R, CCK2R, and other activated class-A GPCRs. **a**, **b** Superposition of SR146131-activated CCK1R (pink) with CCK-8-bound CCK2R (blue). Side view (**a**). Extracellular and cytoplasmic views (**b**). **c** Superposition of SR146131-activated CCK1R (pink) with active β_2_AR (dark green) and inactive β_2_AR (gray). **d–g** Close-up views of conformational changes in crucial residues involved in CCK1R (pink) and β_2_AR (dark green) activation.

Consistent with the great sequence identity (50%) between two CCKRs and the fact that G_q_ protein is the primary coupling effector protein of CCK1R, the overall structures of active CCK1R and CCK2R are similar (root mean square deviation (RMSD) value at Cα of 0.88 Å), with primary differences at the ECLs, TM7–Helix8 hinge, and Helix8 (Fig. 5a, b).

The outward movement of TM6 is a hallmark of receptor activation and the various extent of the TM6 openness relative to other TMs was proposed to be the determinants of G protein subtype specificity^47,48^. Surprisingly, structural superimposition of the CCK1R–G_s_ complex onto the well-studied G_s_-coupled GPCR β_2_AR reveals striking differences regarding the position of TM6, and TM7–Helix8 hinge. CCK1R TM6 locates between the positions of β_2_AR TM6 in the inactive and active states (Fig. 5c). This smaller opening of the intracellular pocket for G protein is not suitable to engage with the G_s_ protein due to potential steric hindrance (Supplementary Fig. S5a). The α5 helix of Gα_s_ protein is bulkier than that of other G proteins (Supplementary Fig. S5b) and thus it is believed that the engagement of G_s_ requires larger outward movement of TM6. Therefore, we hypothesized that the unique conformation of the intracellular half of the active CCK1R would lead to a non-canonical G_s_-coupling mode that is elaborated below. In addition, the positioning of TM6 in G_s_-coupled CCK1R is unexpectedly similar to that in G_i/o_- and G_q_-coupled complexes (Supplementary Fig. S5c, d), suggesting a G_i_-coupling capability of CCK1R which was further confirmed by the cell signaling assay (Supplementary Fig. S5e). This observation also supports the previous hypothesis that the smaller displacement of TM6 in the G_i/o_-bound class-A GPCRs might preclude the binding of G_s_^49^.

The conformations of residues critical for receptor activation, e.g., E^3.49^R^3.50^Y^3.51^, N^7.49^P^7.50^xxY^7.53^, P^5.50^T^3.40^F^6.44^, and the toggle switch motifs, are conformationally similar between the structures of active CCKRs, suggesting a shared activation mechanism, despite that CCK1R and CCK2R exhibit different G protein-coupling profiles (Fig. 5d–g). The CCK2R–G_q_ structure is similar to other reported class-A GPCR–G_q_ complexes regarding the overall conformation of TMD and the positioning of TM6 (Supplementary Fig. S5d).

### Structural basis of G protein coupling of CCKRs

**Fig. 6:**
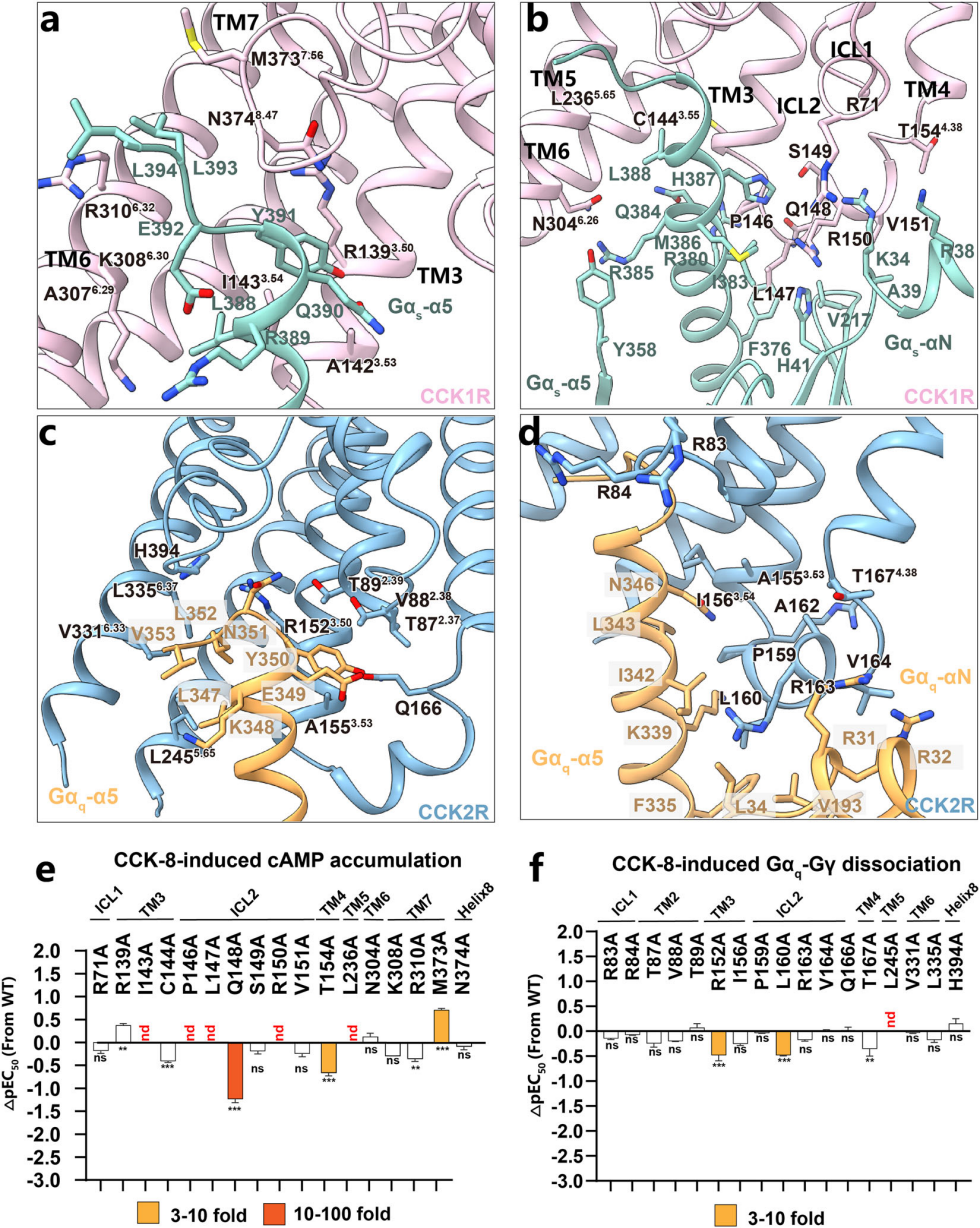
Structural basis of G protein coupling of CCKRs. **a–d** Detailed interactions between CCK1R (pink) and the last seven residues of Gα_s_-α5 helix (cyan) (**a**); between CCK1R (pink) and residues of Gα_s_ excluding α5 helix region (cyan) (**b**); between CCK2R (blue) and the last seven residues of Gα_q_-α5 helix (orange) (**c**); between CCK2R (blue) and residues of Gα_q_ excluding α5 helix region (orange) (**d**). **e** Mutagenesis analysis of residues in the Gα_s_-binding site in CCK1R using CCK-8-induced cAMP accumulation assay. **f** Mutagenesis analysis of residues in the Gα_q_-binding site in CCK2R using CCK-8-induced Gα_q_–Gγ dissociation assay. Bars represent differences in calculated CCK-8 potency (pEC_50_) for representative mutants relative to WT. Data were colored according to the extent of the effect. ns, no significance, **P < 0.001, ***P < 0.0001 (one-way ANOVA followed by Dunnett’s post hoc test, compared with the response of WT). nd, pEC_50_ change went beyond the detection range.

Globally, the structure of the CCK1R–G_s_ complex is similar to the CCK2R–G_q_ complex, reflecting a similarity in the conformation of nucleotide-free states of CCKRs in complex with the G protein. The primary interactions in both complexes occur between ICL2, TM3, TM5, and TM6 on the receptor and the αN, αN–β1 loop, and α5 helix on the Gα subunit of the G protein (Fig. 6a–d). The most striking differences between the CCK1R–G_s_ and CCK2R–G_q_ complexes are in the conformation and relative position of the α5 helix of both G proteins. Strikingly, the displacement of the intracellular tip of TM6 in the CCKR–G protein structures (i.e., CCK1R–G_s_ and CCK2R–G_q_) is smaller than that observed in most other class-A GPCR–G_s_ structures, but similar to the G_i/q_-bound GPCRs (Supplementary Fig. S5a–d, f)^50–59^. Relative to GDP-bound G protein structure in the inactive state^60^, the α5 helix of Gα (Gα_s_ and Gα_q_) in the CCK1R–G_s_ and CCK2R–G_q_ complexes undergoes significant structural rearrangements upon binding to the CCKR intracellular pocket, thus facilitating extensive interactions. In the CCK1R–G_s_ complex, the G_s_ protein formed extensive interactions with ICL2 and TM3–TM7 of CCK1R (Fig. 6a, b). In detail, the last seven amino acids of the Gα_s_-α5 helix connect with R139^3.50^, A142^3.53^, and I143^3.54^ of TM3, L236^5.65^ of TM5, K308^6.30^, A307^6.29^ and R310^6.32^ of TM6, and N374^8.47^ at the TM7–Helix8 hinge in CCK1R (Fig. 6a). In addition, P146, L147, Q148, R150, and V151 at ICL2 also contribute to G_s_ protein recognition, primarily interacting with the αN helix, αN–β1 loop, and α5 helix of the G protein. Over half of the CCK1R and G_s_ interactions cluster in the amino-terminal end of ICL2 and C-terminal end of TM3 (Fig. 6b). Considering that these residues form the major interactions between the receptor and G protein, alanine mutagenesis studies, and functional assays were performed to determine which residues might be key to G protein recognition. Consistent with the structural observations, I143^3.54^A, P146^ICL2^A, L147^ICL2^A, L236^5.65^A, and R150^ICL2^A mutations abolished the potency of G_s_ signaling in the cAMP accumulation assay (Fig. 6e; Supplementary Table S2). Of note, CCK1R can also couple with the G_q_ protein through this common pocket (Supplementary Fig. S1b), whereas CCK2R couples with G_q_ but not with the G_s_ protein^36^. Similarly, in the CCK2R–G_q_ complex, the last seven amino acids of the G_q_-α5 helix extensively interacts with V88^2.38^ and T89^2.39^ of TM2, R152^3.50^, A155^3.53^, and I156^3.54^ of TM3, Q166^ICL2^ at ICL2, L245^5.65^ of TM5, V331^6.33^ and L335^6.37^ of TM6, and H394^8.47^ at the TM7–Helix8 hinge in CCK2R (Fig. 6c). Other interactions between G_q_ and CCK2R are most in ICL2 of CCK2R (Fig. 6d). Among them, the mutagenesis NanoBiT assay showed that L245^5.65^A abolished the G_q_ signaling capability of CCK2R, suggesting that L245^5.65^ plays a key role in G_q_ activation. In addition, the R152^3.50^A mutation decreased the efficacy of G protein signaling to 30% and the potency by over 3-fold that of the wild-type, while I156^3.54^A, Q166^ICL2^A, and L335^6.37^A reduced CCK-8 efficacy by 50% but did not affect the potency (Fig. 6f; Supplementary Table S3). Additionally, superimposing the three activated structures (i.e., CCK1R, CCK2R, and β_2_AR) reveals that, the residues at 5.65 in CCKRs are L236 and L245, respectively, while it is A226 in β_2_AR. The bulkier leucine residues in CCK1R and CCK2R push the α5 helix of G_s_ and G_q_ further close to the TM3–ICL2–TM4 half. Mutagenesis and functional experiments suggest that L^5.65^ is crucial for receptor activation for both CCKRs, evidenced by the complete loss of agonist activity by L^5.65^A (Supplementary Fig. S5g).

### G protein subtype specificity between CCK1R and CCK2R

**Fig. 7:**
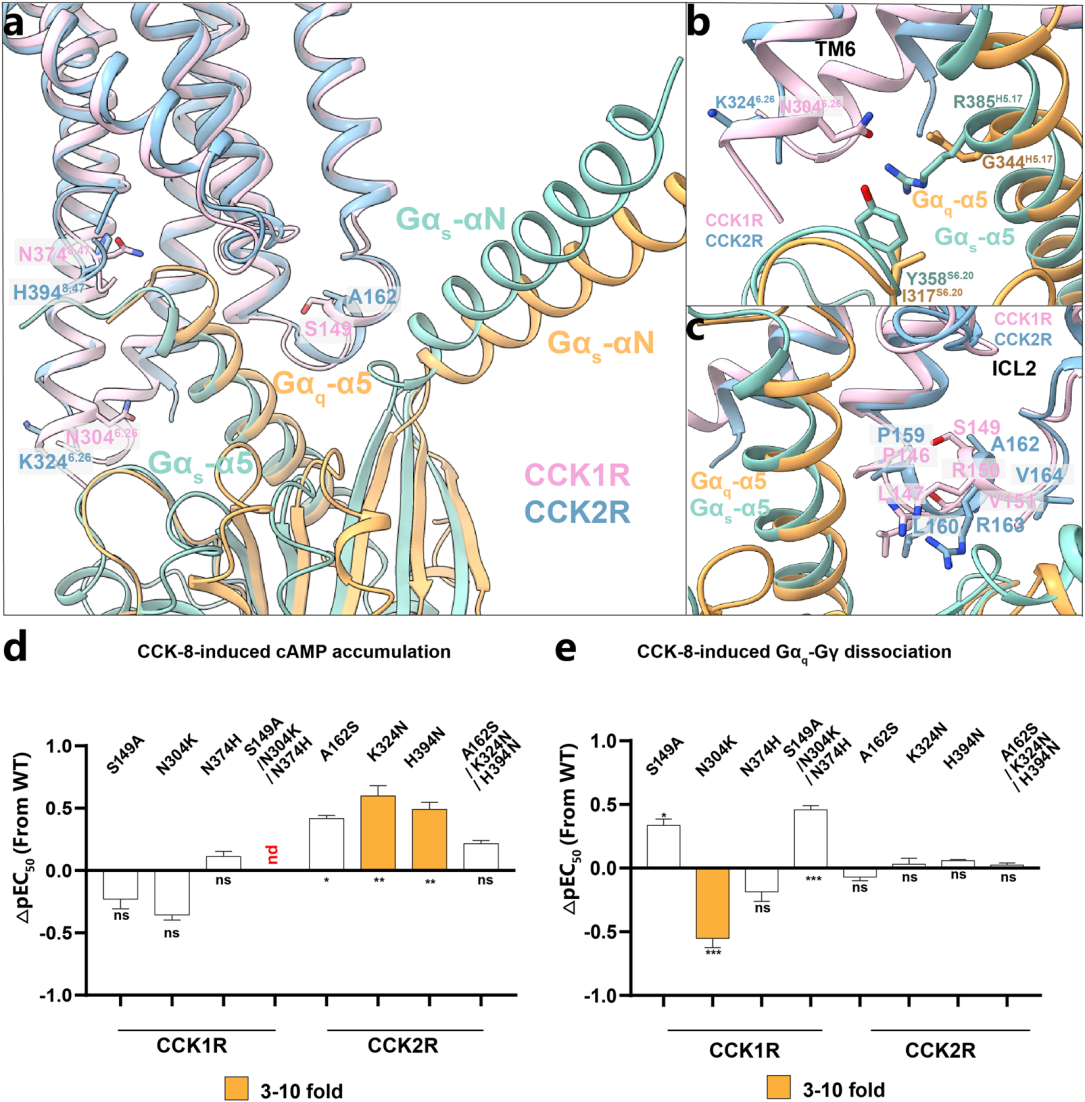
G protein subtype specificity between CCK1R and CCK2R. **a** Comparison of G protein conformations in SR146131–CCK1R (pink)–Gα_s_ (cyan) and CCK-8–CCK2R (blue)–Gα_q_ (orange) structures. **b** Interactions between N304^6.26^ in CCK1R (pink) and R385^H5.17^ and R358^S6.20^ in Gα_S_ (cyan). The corresponding residues in CCK2R (blue) and Gα_q_ (orange) are also shown. **c** Detailed interactions between ICL2 of CCK1R (pink) and Gα_s_ (cyan) and between ICL2 of CCK2R (blue) and Gα_q_ (orange). **d**, **e** Residues involved in G protein coupling in CCK1R/CCK2R are respectively substituted by the corresponding residues in CCK2R/CCK1R, and the effect of each single- or triple-mutation on CCK-8-induced cAMP accumulation (**d**) or CCK-8-induced Gα_q_–Gγ dissociation (**e**) is shown.

Given that the G protein-binding pockets of CCK1R and CCK2R exhibit extreme similarity in terms of pocket shape and amino acid sequence (Fig. 7a–c; Supplementary Fig. S6a), it is surprising that their G protein-coupling profiles are different. In detail, both CCK1R and CCK2R could couple to G_q_, whereas only CCK1R exhibits the G_s_-coupling capability. Our CCK1R–G_s_ and CCK2R–G_q_ structures could provide a unique opportunity to understand the molecular basis for G protein-coupling specificity, at least for CCKRs. We compared the CCK1R–G_s_ complex with other GPCR–G_s_ protein complexes. In each panel (Supplementary Fig. S5a, f), the complexes were aligned by the receptor, thus showing differences in the orientation of the G_s_ protein relative to the receptor. The αN orientation of CCK1R–G_s_ is closest to that of A_2A_R–G_s_ and β_2_AR–G_s_, whereas the conformation of the α5 helix shows marked differences when compared to that in other GPCR–G_s_ complexes. Owning to the unique transducer–pocket conformation, the extreme C-terminus of G_s_-α5 helix unexpectedly inserts into the cleft between TM6 and the TM7–H8 hinge (Supplementary Fig. S5b). In the CCK2R–G_q_ complex, G_q_ undergoes an anticlockwise rotation relative to the receptor compared to G_s_ (except for EP4R–G_s_) (Supplementary Fig. S5a, f). This difference makes the α5 helix of G_q_ moving closer to the TM2–TM3–TM4 half (Supplementary Fig. S5h, i).

Overall, the binding pockets of the G proteins of CCK1R and CCK2R were similar in sequence and conformation (Fig. 7a; Supplementary Fig. S6a). However, compared to CCK2R, TM6 of CCK1R is closer to Gα_s_, leading to a connection between G_s_ (Y358^S6.20^ and R385^H5.17^) and the CCK1R residue N304^6.26^, whereas the corresponding residue in CCK2R (K324^6.26^) could potentially form a repulsive force against Gα_s_ (Fig. 7b). Therefore, the substitution of K324^6.26^ to N in CCK2R resulted in an increase of the coupling potency with G_s_ by over threefold, which supports that N304^6.26^ contributes partially to G_s_ coupling of CCK1R (Fig. 7d; Supplementary Tables S2, S6).

The interaction surfaces between ICL2 and Gα_s/q_ in both receptors are also similar, with the buried surfaces between ICL2 of CCK1R and Gα_s_, and ICL2 of CCK2R and Gα_q_ accounting for 49% and 44% of the total surface of the corresponding Gα subunit, respectively (Fig. 7c; Supplementary Fig. S7a, b). A conserved motif PL^34.51^QXR^34.54^VWT is present in CCKR ICL2s (Figs. 6b, d, 7c). The counterparts of the Gα_s/q_ side on the interfaces between ICL2 and Gα_s/q_ are conserved as well, involving L34^S1.02^, V193^S3.01^, F335^H5.08^, K339^H5.12^, and I342^H5.15^ of Gα_q_ via hydrophobic interactions and H41^S1.02^, V217^S3.01^, F376^H5.08^, R380^H5.12^, and I383^H5.15^ of Gα_s_. H41^S1.02^ of Gα_s_ forms an additional hydrogen bond with R^34.54^ in CCK1R ICL2 (Fig. 6b, d). We conducted the alanine mutagenesis studies and performed a cAMP accumulation assay (G_s_ signaling) and NanoBiT assay (G_s_ and G_q_ signaling) to determine the role of ICL2s in G_s/q_ coupling. Mutations of several residues in CCK1R ICL2, including P146^34.50^, L147^34.51^, and R150^34.54^, dramatically attenuated receptor–G_s_ coupling than G_q_ coupling, as measured by cAMP accumulation assay and NanoBIT assay (Fig. 6e; Supplementary Fig. S7c, d), implying that ICL2 of CCK1R is crucial for G_s_ binding but not for G_q_.

Within the conserved intracellular cavity for G protein coupling, three pairs of residues are non-conserved, including S149/A162^ICL2^, N304/K324^6.26^, and N374/H394^8.47^ (S149, N304, and N374 in CCK1R; A162, K324, and H394 in CCK2R). Although the single-residue mutations of CCK1R to the corresponding residue in CCK2R (i.e., S149A^ICL2^, N304K^6.26^, and N374H^8.47^) caused little change to CCK1R-mediated G_s_ signaling, surprisingly, the triple-mutation of CCK1R (S149A^ICL2^/N304K^6.26^/N374H^8.47^) completely abolished G_s_ signaling while only slightly affecting G_q_ signaling of CCK1R, indicating that the combination of three amino acids collectively contributes to the selectivity of CCKRs towards G_s_ or G_q_ for CCKR family, especially essential for the G_s_ coupling of CCK1R (Fig. 7d, e; Supplementary Fig. S7e and Tables S3, S7). To test this hypothesis, we mutated the non-conserved three residues in CCK2R to the corresponding counterparts in CCK1R and tested whether these mutations could restore G_s_ coupling for CCK2R. We found that the single-residue mutations (A162S ^ICL2^, K324N^6.26^, and H394N^8.47^) in CCK2R increased the potency for G_s_ protein coupling, consistent with our hypothesis that these three residues contribute to the G_s_ coupling of CCK1R.

## Discussion

In this paper, we reported four high-resolution cryo-EM structures of CCK1R and CCK2R signaling complexes. The structures, together with mutagenesis studies, revealed distinct features of the CCK1R and CCK2R ligand-binding pockets that determine CCK1R selectivity for sulfated CCK-8 and SR146131, and CCK2R selectivity for gastrin-17, and thus highlighted the activation mechanism for both CCK1R and CCK2R. Sulfation of CCK and gastrin in the body is highly regulated by sulfotransferase (SULT) enzymes with tissue-specific expression patterns, e.g., SULT4 is predominantly expressed in the brain^61^. The sulfation level of CCK and gastrin is also correlated with sub-cellular and cellular distribution. Interestingly, the non-sulfated CCK is enriched in neuronal cell bodies, whereas the sulfated CCK predominantly distributes in the nerve terminals^62^. CCKRs differentially recognize the sulfated and non-sulfated ligands enabling subtle and precisely regulated responses to extracellular stimuli. The orthosteric binding pockets for CCK and gastrin in CCKR family members are generally similar in topology and conserved in amino acid sequence, allowing uniform recognition of the strictly conserved last five residues in the extreme C-terminus of peptide ligands. The unique binding mode of gastrin in CCK2R permits its selective activation of CCK2R, which is also known as the gastrin receptor. Last, the ECL2 lid possesses a key and exclusive residue R197^ECL2^ in CCK1R, leading to a positively charged sub-pocket for TYS. Therefore, additional polar interactions with sulfated ligand-mediated by R197^ECL2^ significantly enhance the affinity and potency, providing an explanation for the observation that CCK1R prefers sulfated CCK while CCK2R binds to the sulfated and non-sulfated ligands equally well.

Our study also provides a structural basis for the distinct G protein-coupling specificity of CCK1R and CCK2R. The amino acid sequence of Gα_s_ in the α5 helix region is closely conserved with that in Gα_q_, while showing a significant difference from that of Gα_i/o_ (Supplementary Fig. S6b). Although recent advances in the cryo-EM technique facilitate structure determination of a vast of GPCR–G protein complexes, it remains elusive how GPCRs achieve G protein-coupling specificity between G_s_ and G_q_ protein subtypes. Notably, we observed a conserved motif P^34.50^L^34.51^XXR^34.54^ in CCKR ICL2s, which is necessary for G_s_ coupling but surprisingly not for G_q_ signaling. Thus, we propose that ICL2 plays a key role in G protein engagement and coupling selectivity determination for the CCKR family. CCK2R contains the PLXXR motif in ICL2 and has evolved three residue substitutions compared with CCK1R, including S149/A162^ICL2^, N304/K324^6.26^, and N374/H394^8.47^ (S149, N304, and N374 in CCK1R; A162, K324, and H394 in CCK2R), to disable its G_s_ coupling.

Together, our results provide unprecedented structural insights into the pharmacology and signaling of CCK1R and CCK2R and multiple structural templates for rational drug design targeting the CCKRs. Furthermore, gut–brain signaling, the common mechanism by which these two receptors exert their functions, could also be targeted for the treatment of not only metabolic diseases such as obesity and metabolic syndrome but also neuropsychiatric disorders such as anxiety and pain. Our structures of CCK1R and CCK2R will set the stage for future efforts to develop novel therapeutic strategies to selectively target CCK2R to fight anxiety, depression, pain, and possibly other emotional diseases without causing side effects in the intestinal tract.

## Materials and methods

### Experimental model and subject details

*Spodoptera frugiperda* (Sf9, Expression systems) cells were grown in ESF 921 medium at 27 °C and 120 rpm. HEK293T cells were grown in a humidified incubator with 5% CO_2_ at 37 °C using a medium supplemented with 100 I.U./mL penicillin and 100 mg/mL streptomycin (Invitrogen). The human HEK293T cells were maintained in DMEM (VWR) containing 10% fetal bovine serum (FBS, VWR).

### Constructs

The wild-type human CCK1R and CCK2R with C-terminal 15-residue depleted were subcloned into pFastBac vector with an N-terminal FLAG-tag and C-terminal 10× His-tag. To obtain a receptor–G protein complex with good homogeneity and stability, we used the NanoBiT tethering strategy, in which the C-terminus of rat Gβ_1_ was linked to the HiBiT subunit with a 15-amino acid polypeptide (GSSGGGGSGGGGSSG) linker and the C-termini of CCK1R and CCK2R were directly attached to LgBiT subunit followed by a TEV protease cleavage site and a double MBP-tag. A dominant-negative human Gα_s_ (DNGα_s_) was generated by site-directed mutagenesis as previously described to limit G protein dissociation. Twenty-nine amino acids at the N-terminus of wild-type Gα_q_ were replaced by the corresponding sequence in Gα_i1_ to facilitate the binding of scFv16. The constructs were cloned into both pcDNA3.1 and pFastBac vectors for functional assays in mammalian cells and protein expression in insect cells, respectively. All modifications of the receptor had no effect on ligand binding and receptor activation. Other constructs including the full-length and various site-directed mutated human CCK1R and CCK2R were cloned into the pcDNA3.1 vector for cAMP accumulation and NanoBiT–G protein dissociation assay.

### Expression, complex formation, and purification

CCK1R, DNGα_s_, and Gβγ-peptide were co-expressed in Sf9 insect cells (Expression System) while the CCK2R, G_qiN_, Gβγ-peptide, Ric-8A were co-expressed in Sf9 insect cells, using the Bac-to-Bac baculovirus expression system (Thermo Fisher). Cell culture was collected by centrifugation 48 h post-infection and stored at −80 °C until use. The sediment was re-suspended in 20 mM HEPES, pH 7.5, 100 mM NaCl, 2 mM MgCl_2_, 50 mU/mL Apyrase (Sigma), and 100 mM ligand (CCK-8 or SR146131 or gastrin-17). After incubation at room temperature for 1.5 h, the membranes were solubilized by the addition of 0.5% (w/v) Lauryl Maltose Neopentylglycol (LMNG, Anatrace) and 0.1% (w/v) Cholesteryl Hemisuccinate TRIS salt (CHS, Anatrace) for 2 h at 4 °C. The supernatant was isolated by centrifugation at 30,000× *g* for 30 min and then incubated for 1 h at 4 °C with pre-equilibrated MBP resin. After binding, the resin was washed with 15 column volumes of 20 mM HEPES, pH 7.5, 100 mM NaCl, 2 mM MgCl_2_, 0.01% (w/v) Lauryl Maltose Neopentylglycol (LMNG, Anatrace), 0.002% (w/v) CHS, and 10 mM ligand. The complex was eluted with 5 column volumes of 20 mM HEPES, pH 7.5, 100 mM NaCl, 2 mM MgCl_2_, 0.01% (w/v) LMNG, 0.002% (w/v) CHS, 10 mM Maltose and 10 mM ligand. The protein was then concentrated and loaded onto a Superdex 200 Increase 10/300 column (GE Healthcare) pre-equilibrated with buffer containing 20 mM HEPES, pH 7.5, 100 mM NaCl, 0.00075% (w/v) LMNG, 0.00025% (w/v) glyco-diosgenin (GDN, Anatrace), 0.0002% (w/v) CHS and 10 mM ligand. The fractions for the monomeric complex were collected and concentrated for electron microscopy experiments.

### Cryo-EM grid preparation and data collection

For the cryo-EM grid preparation, 3 μL purified CCK-8–CCK1R–G_s_–Nb35 complex at the concentration of ~15 mg/mL, SR146131–CCK1R–G_s_–Nb35 complex at the concentration of 13 mg/mL, CCK-8–CCK2R–G_q_–scfv16 complex at the concentration of 11 mg/mL, and gastrin-17-CCK2R–G_q_–scFv16 complex at the concentration of 13 mg/mL were applied individually to a glow discharged holey carbon EM grid (Quantifoil, Au300 R1.2/1.3) in a Vitrobot chamber (FEI Vitrobot Mark IV). Protein concentration was determined by absorbance at 280 nm using a Nanodrop 2000 Spectrophotometer (Thermo Fisher Scientific). The Vitrobot chamber was set to 100% humidity at 4 °C. The sample-coated grids were blotted before plunge-freezing into liquid ethane and stored in liquid nitrogen for data collection. The microscope was operated at 300 kV accelerating voltage, at a nominal magnification of 29,000 in counting mode, corresponding to a pixel size of 1.014 Å.

### Image processing and map reconstruction

Dose-fractionated image stacks were subjected to beam-induced motion correction using MotionCor2.1^63^. A sum of all frames, filtered according to the exposure dose, in each image stack was used for further processing. Contrast transfer function (CTF) parameters for each micrograph were determined by Gctf v1.06^64^. Particle selection, 2D, and 3D classifications were performed on a binned dataset with a pixel size of 2.028 Å using RELION-3.0^65^.

For the CCK-8–CCK1R–G_s_–Nb35 complex, auto-picking-yielded 5,768,688 particle projections were subjected to 3D classification to discard particles in poorly defined classes, producing 1,608,563 particle projections. The particles were extracted and subjected to 3D classification on the complex, and the best-resolved class was selected. The resulting set of 299,092 particles was subjected to CTF refinement, Bayesian polishing and 3D auto refinement using the pixel size of 1.014 Å. The final map has an indicated global resolution of 3.2 Å at a Fourier shell correlation (FSC) of 0.143.

For the SR146131–CCK1R–G_s_–Nb35 complex, auto-picking-yielded 5,213,984 particle projections were subjected to 3D classification to discard particles in poorly defined classes, producing 898,915 particle projections. The re-picked particles were extracted and subjected to two rounds of 3D classification with four classes; the best-resolved class was selected each time. The resulting set of 606,684 particles was subjected to CTF refinement, Bayesian polishing and 3D auto refinement using the pixel size of 1.014 Å. The final map has an indicated global resolution of 3.0 Å at an FSC of 0.143.

For the CCK-8–CCK1R–G_s_–Nb35 complex, auto-picking yielded 4,302,863 particles. Among them, 669,008 particles presented better density in ECD and G protein region than the other. This subset was subjected to 3D classification with a mask focused on the complex and the receptor. Finally, 484,441 particles were subjected to 3D refinement with a mask on the complex and Bayesian polishing with a pixel size of 1.014. The final map has an indicated global resolution of 3.1 Å at an FSC of 0.143.

For the gastrin-17–CCK2R–G_q_–scFv16 complex, a total of 3,995,739 particles were automatically picked from 6611 images. A dataset of 551,048 particles was subjected to 3D refinements, yielding a final map with a global nominal resolution at 3.1 Å by the 0.143 criteria of the gold-standard FSC. Half-reconstructions were used to determine the local resolution of each map.

### Structure model building and refinement

The building of a model for the CCK-8–CCK1R–G_s_–Nb35 complex was aided by the quality and resolution of our map. The initial template of CCK1R was obtained from the GPCR Database, and the heterotrimeric G protein was derived from active-state PTH1R (PDB ID: 6NBF) with the receptor removed. The structure of the SR146131–CCK1R–G_s_–Nb35 complex was generated by the fitting of the CCK-8–CCK1R–G_s_–Nb35. For the structure of the CCK-8–CCK2R–G_q_–scFv16 and gastrin-17–CCK2R–G_q_–scFv16 complexes, the initial template of CCK2R was also from the GPCR Database, and 5-HT–G_q_ (PDB ID: 6WHA) was used as an initial template for Gα_q_ and Gβγ model building. Models were docked into the EM density map using UCSF Chimera. This starting model was then subjected to iterative rounds of manual adjustment and automated refinement in Coot and Phenix, respectively. The final refinement statistics were validated using the module comprehensive validation (cryo-EM) in PHENIX. Structural figures were prepared in Chimera, Chimera X, and PyMOL (https://pymol.org/2/). The final refinement statistics are provided in Supplementary Table 1.

### CCK1R and CCK2R G_s_-mediated G_s_-cAMP accumulation assay

CCK1R and CCK2R G_s_-mediated G_s_-cAMP accumulation assays were performed using HEK293T cells (ATCCCRL-11268) transiently expressing human CCK1R and the cAMP biosensor GloSensor-22F (Promega). Cells were seeded (4 × 10^3^ cells, 40 μL/well) into 384-well culture plates and incubated for 24 h at 37 °C in 5% CO_2_. Next day, the culture medium was removed and the equilibration medium containing 4% (v/v) dilution of the GloSensor™ cAMP reagent stock solution was added to each well. To obtain the concentration-response curves, serially diluted agonists were added to each well to stimulate the cells. The luminance signal was measured using 0.5-s intervals after ligand addition (TECAN, 25 °C). Concentration-responses were generated from the peak response. cAMP accumulation was analyzed by a standard dose-response curve using GraphPad Prism 8.0 (GraphPad Software). EC_50_ and pEC_50_ ± SEM were calculated using nonlinear regression (curve fit). Data were means ± SEM from at least three independent experiments performed in technical triplicates. Span, sample size, and expression are provided in Supplementary Tables S2, S5, and S6. Dose-response curves are shown in Supplementary Fig. S8.

### NanoBit–G protein dissociation assay

G protein activation was measured by a NanoBiT–G protein dissociation assay in which GPCR-induced G protein dissociation is monitored by a NanoBiT system. A large fragment (LgBiT) of the NanoBiT luciferase was inserted into the helical domain (between the αA and the αB helices) of a Gα subunit with 15-amino acid flexible linkers. A small fragment (SmBiT) was N-terminally fused to a C68-mutated Gγ2 subunit with a 15-amino acid flexible linker. HEK293T cells were seeded in a six-well plate and allowed to grow to 80% confluence before transfection. For measuring G_q_ signaling, a plasmid mixture consisting of 200 ng LgBiT-inserted Gα_q_ subunit, 500 ng Gβ1, 500 ng C68S-mutant SmBiT-fused Gγ2 (C68S), 200 ng cholinesterase-8A (Ric-8A) and 400 ng test GPCR in 200 μL of Opti-MEM (Gibco) was transfected into cells using polyethylenimine (PEI). For measuring G_s_ signaling, a plasmid mixture consisting of 200 ng LgBiT-inserted Gα_s_ subunit, 1000 ng Gβ1, 1000 ng C68S-mutant SmBiT-fused Gγ2 (C68S), 400 ng test GPCR in 200 μL of Opti-MEM (Gibco) was transiently transfected with PEI. After 1-day transfection, transfected cells were plated onto a 96-well plate treated by cell adherent reagent (Applygen). After 12 h, cells were washed with D-PBS to remove the complete medium and loaded with 40 μL of 10 μM coelenterazine 400a (Maokangbio) diluted in the assay buffer (HBSS containing 0.01% bovine serum albumin and 5 mM HEPES, pH 7.5) per well. After 1-h incubation at room temperature, the plate was measured for baseline luminescence (TECAN). CCK-8 or gastrin-17 at different concentrations (10 μL) was added and incubated for 3–5 min at room temperature before the second measurement. Luminescence counts were normalized to the initial count and fold-change signals over treatment of the lowest CCK-8 or gastrin-17 concentration were used to show G protein dissociation response. Span, sample size, and expression are provided in Supplementary Tables S3, S4, S7. Dose-response curves are shown in Supplementary Fig. S9.

### Receptor expression ELISA

Transiently transfected HEK293T cells were seeded in a poly-d-lysine-coated 96-wells plate for 24 h at 37 °C and 5% CO_2_. In the case of determination of surface receptor expression levels, cells were washed with 1× PBS and fixed with 4% paraformaldehyde for 10 min. Following fixation, cells were blocked with blocking buffer (1% (w/v) BSA/PBS) for 1 h at room temperature. Afterward, cells were incubated with a 1:10,000 dilution of anti-FLAG M2 HRP-conjugated monoclonal antibody (Sigma-Aldrich) in a blocking buffer for another 0.5 h at room temperature. Then, wells were washed three times with blocking buffer and three times with 1× PBS in order. Finally, antibody binding was detected using 80 μL/well diluent SuperSignal Elisa Femto Maximum Sensitivity Substrate (Thermo Fisher Scientific). The luminance signal was measured using 800-ms intervals. Data were analyzed using GraphPad Prism version 8.0.

### Figure preparation

The density maps were prepared in UCSF Chimera (https://www.cgl.ucsf.edu/chimera/) and UCSF Chimera X (https://www.cgl.ucsf.edu/chimerax/). Structural comparison and alignment figures were prepared with PyMOL (https://pymol.org/2/).

## Supplementary information

Supplementary materials

## Data Availability

The atomic coordinates and the electron microscopy maps of the CCK-8– CCK1R–G_s_, SR146131–CCK1R–G_s_, gastrin-17–CCK2R–G_q_, and CCK-8–CCK2R–G_q_ complexes have been deposited in the Protein Data Bank under accession codes 7XOU, 7XOV, 7XOW, 7XOX, and Electron Microscopy Data Bank under accession codes EMD-33359, EMD-33360, EMD-33361, EMD-33362, respectively. All relevant data were included in the manuscript or Supplementary information.
