# Supplementary material for: Structural insights into human brain–gut peptide cholecystokinin receptors
Source: Cell Discov. 2022 Jun 7;8:55. doi: 10.1038/s41421-022-00420-3 (PMC9174195; doi:10.1038/s41421-022-00420-3)
Supplement: Supplementary file 1 — Supplementary materials [file 41421_2022_420_MOESM1_ESM.pdf]

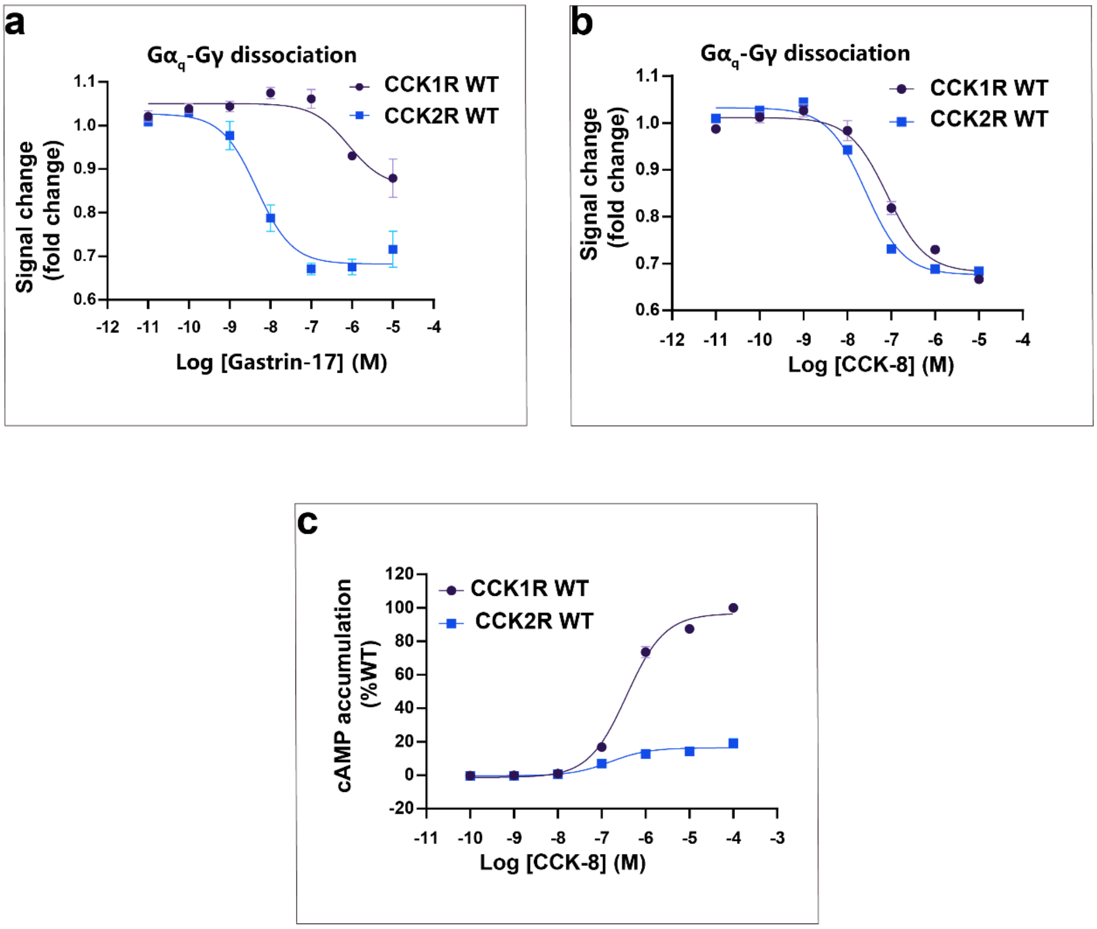

Supplementary Fig. S1 G_q and G_s signaling of CCK1R and CCK2R. a,b Dose response curves of gastrin-17 (**a**) and CCK-8 (**b**) induced $G\alpha_q$–Gγ dissociation using wild type CCKRs were measured by the NanoBiT assay. **c** Dose response curves of CCK-8 induced cAMP accumulation using wild type CCKRs were measured by the Glosensor assay. All data are presented as mean values ± standard error of measurement (SEM).

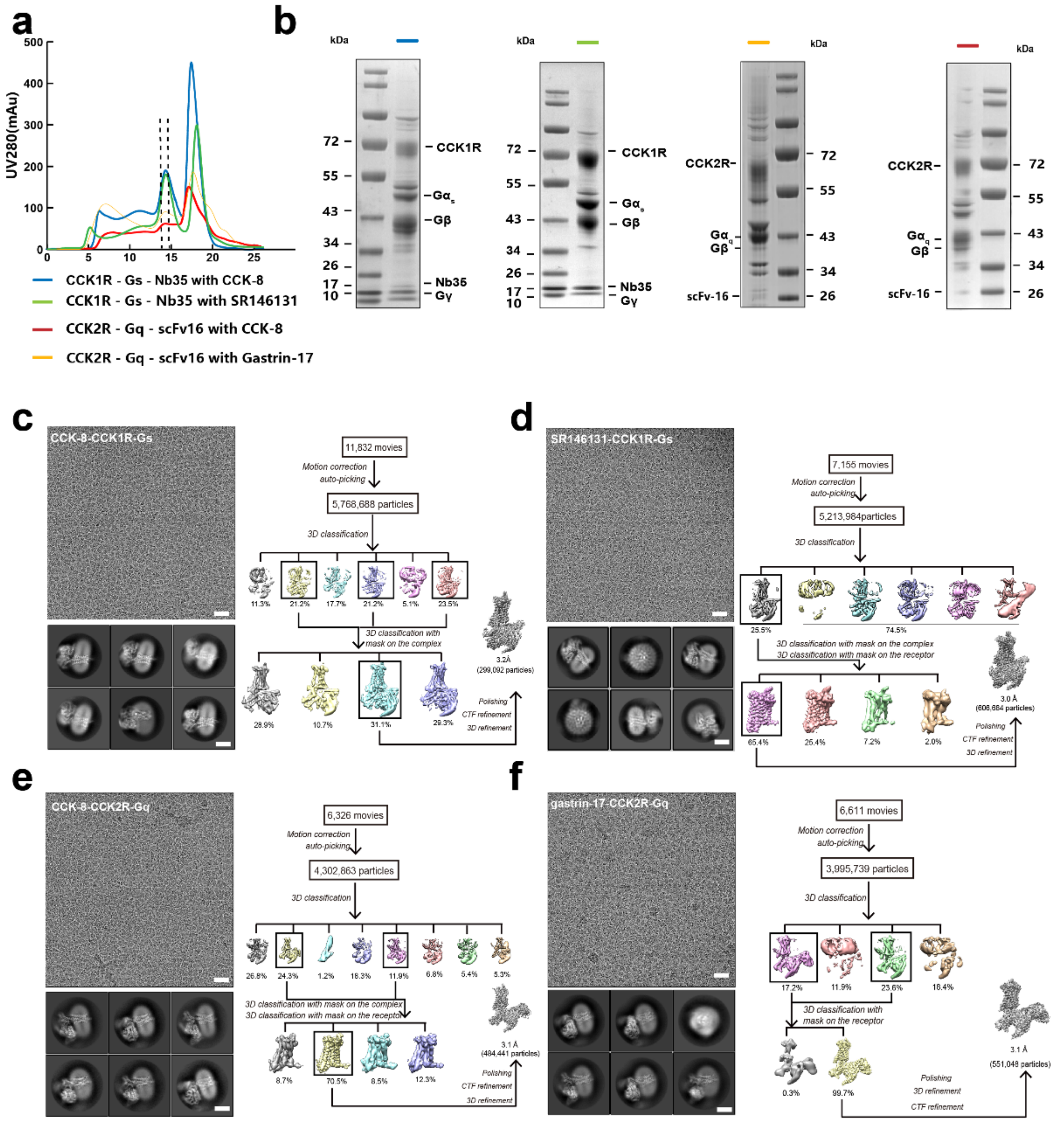

Supplementary Fig. S2 Purification and single-particle reconstructions of agonist-bound CCK1R–G$_s$ and CCK2R–G$_q$ complexes. a,b Size exclusion chromatography (SEC) profile (**a**) and SDS-PAGE analysis (**b**) of CCK-8 and SR146131-–stimulated CCK1R–G$_s$ complexes, and CCK-8 and gastrin-17–stimulated CCK2R–G$_q$ complexes, respectively. Fractions between two dashed lines in SEC profile were pooled and concentrated for cryo-EM analysis. **c-f** Cryo-EM micrographs (scale bar: 30 nm), 2D class averages (scale bar: 5 nm),

and flow chart of cryo-EM data processing for CCK-8 (**c**) and SR146131 (**d**)–bound CCK1R–G_s complexes, and CCK-8 (**e**) and gastrin-17 (**f**)–bound CCK2R–G_q complexes, respectively.

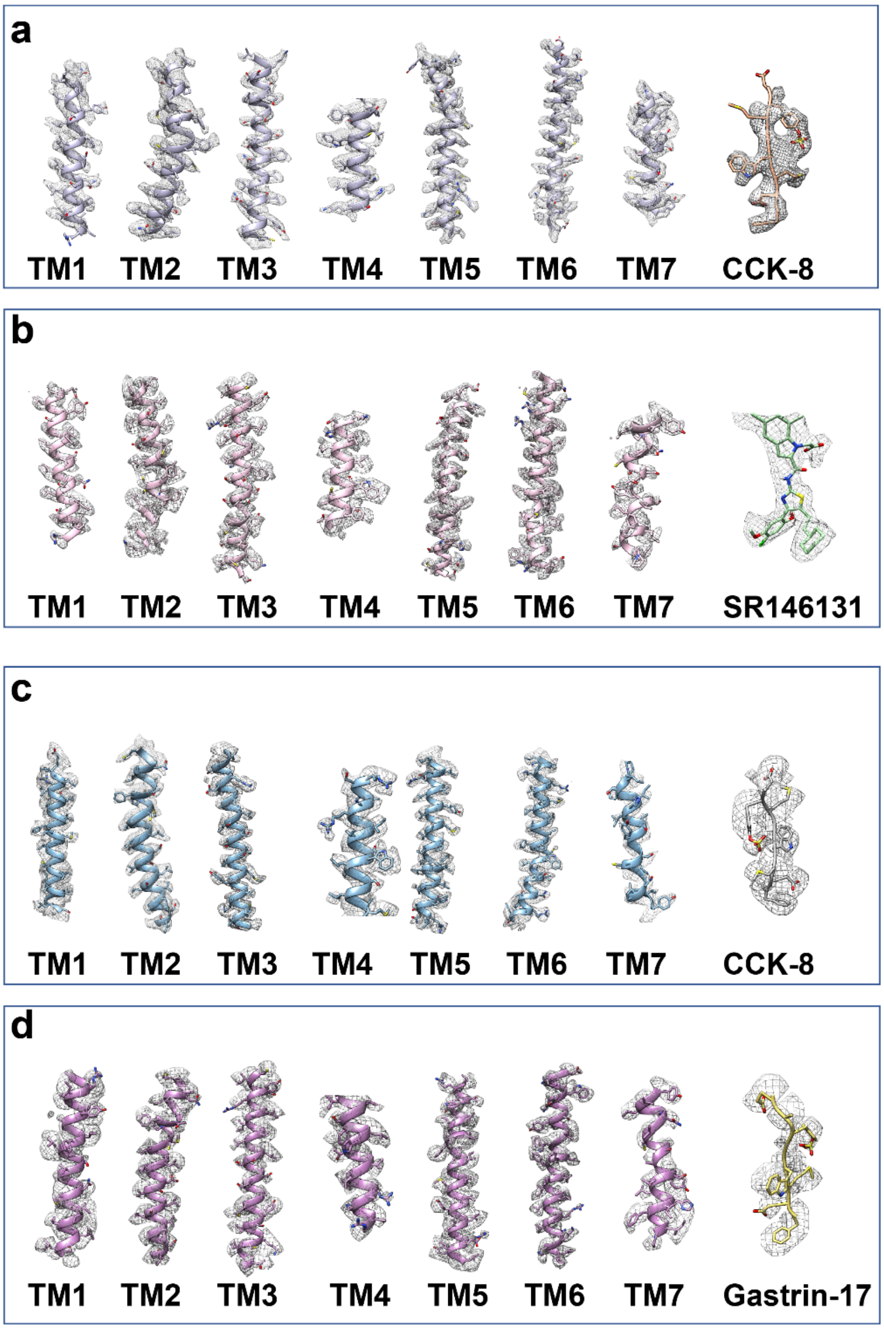

Supplementary Fig. S3 Overall resolution and cryo-EM density analysis of CCK1R–G_s and CCK2R–G_q complexes. a-d EM density maps and models for all seven transmembrane helices and agonists of CCK-8–(**a**) and SR146131–(**b**) bound CCK1R–G_s complexes, and CCK-8–(**c**) and gastrin-17–(**d**) bound CCK2R–G_q complexes. CCK-8-bound CCK1R, light slate blue; CCK-

8, sienna; SR146131-bound CCK1R, pink; SR146131, dark sea-green; CCK-8–bound CCK2R, blue; CCK-8, gray; gastrin-17–bound CCK2R, purple; gastrin-17, yellow.

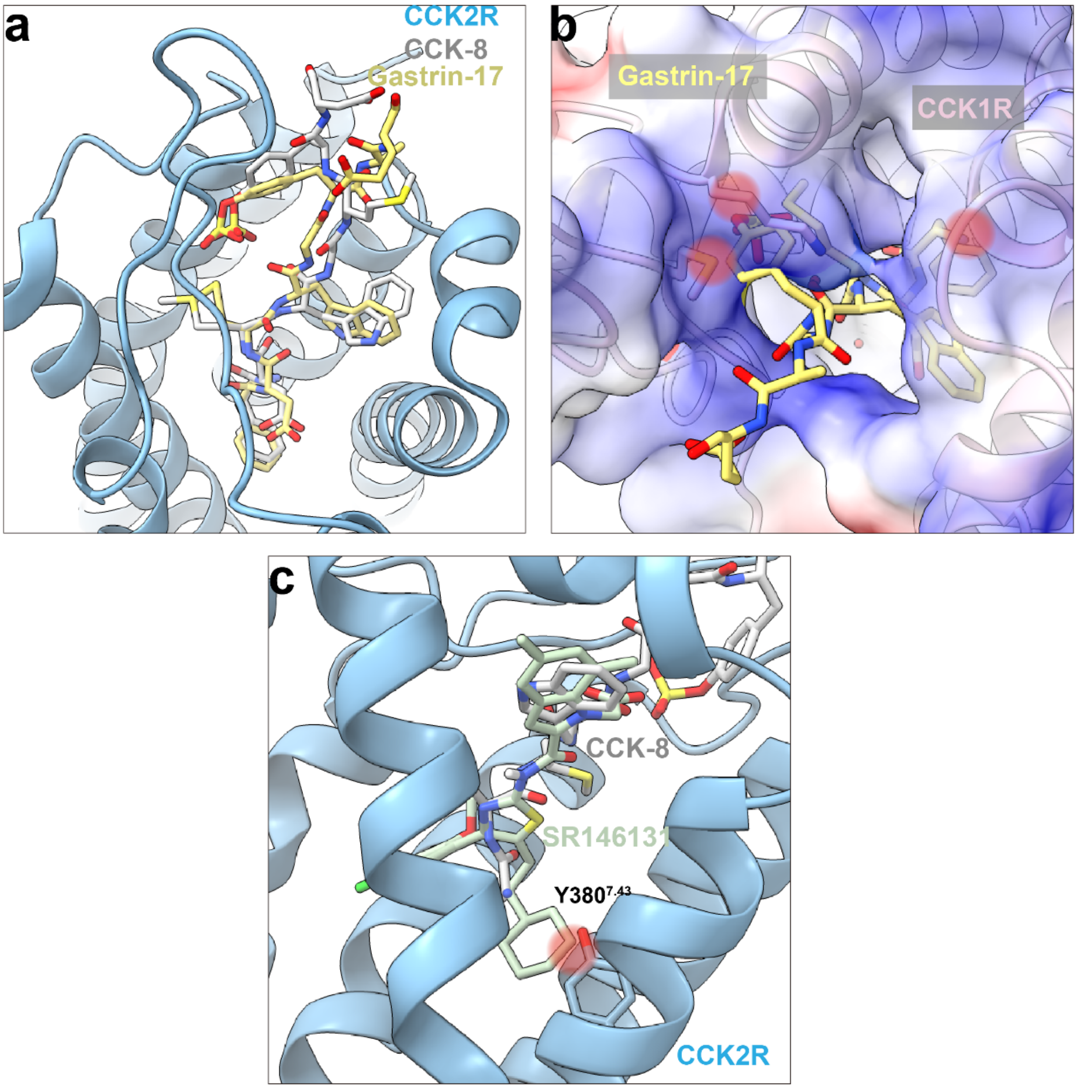

Supplementary Fig. S4 Ligand-binding pocket of CCK1R and CCK2R. a Superposition of CCK-8 (gray)–CCK1R and gastrin-17(yellow)–CCK2R structure. **b** Superposition of gastrin-17(yellow)–CCK2R and CCK-8–CCK1R (pink) structure. Potential steric hindrance is colored in cycle of red. **c** Superposition of CCK-8 (gray) and SR146131 (dark sea-green) in CCK2R. Potential steric hindrance is colored in cycle of red.

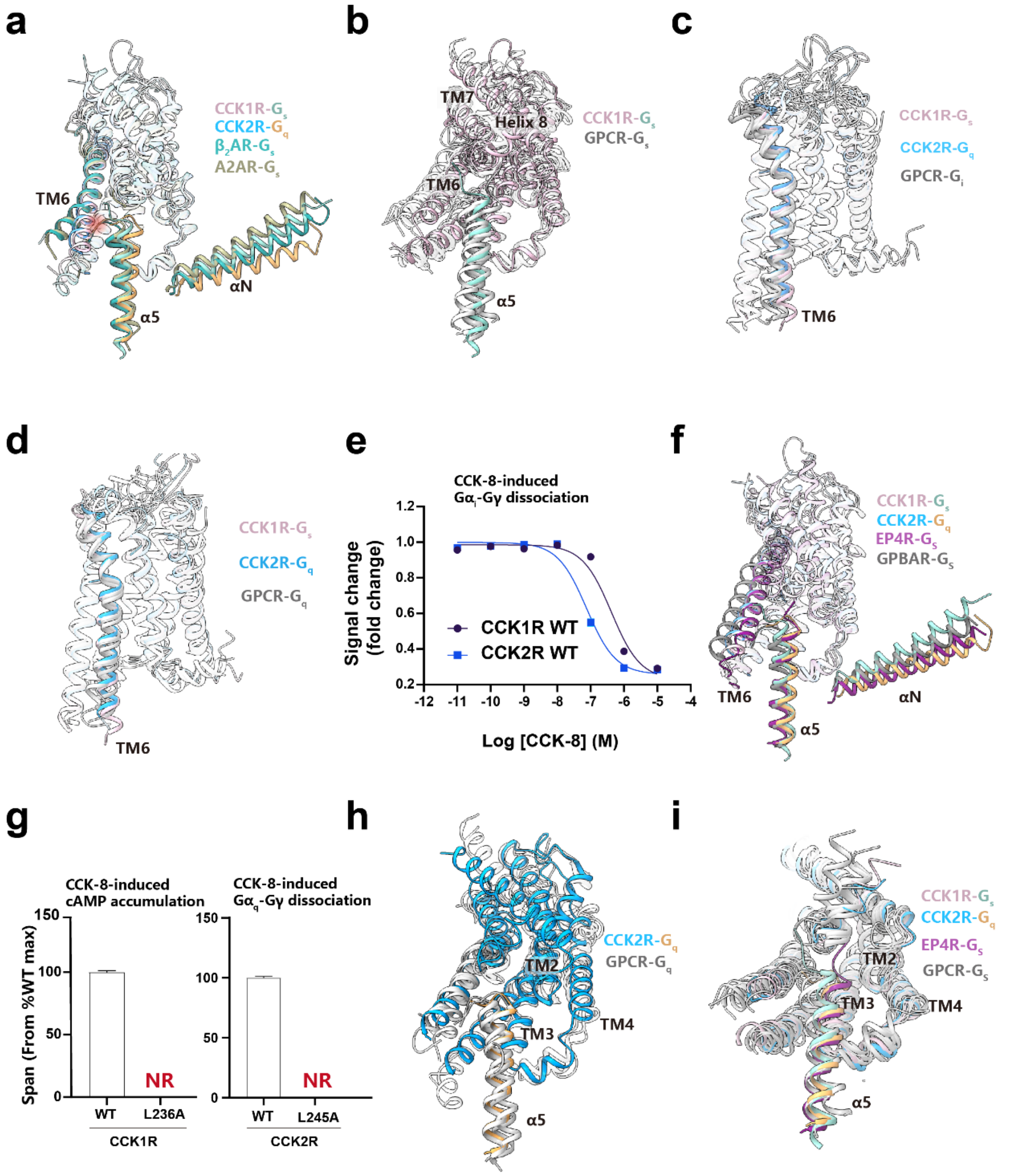

Supplementary Fig. S5 Structural comparisons of active structures of class A receptors and peptide receptors. a Structural superposition of CCK1R (pink) and CCK2R (blue) with β_2AR–G_s (dark green) and A2AR–G_s (dark olive green), potential steric hindrance is colored in cycle of red. **b** Structural superposition of CCK1R (pink) with GPCR–G_s (grey). **c, d** Structural superposition of CCK1R (pink) and CCK2R (blue), with GPCR–G_i (grey) (**c**),

and GPCR–G_q (grey) (**d**). **e** Dose response curves CCK-8 induced $G\alpha_i$–$G\gamma$ dissociation using wild type CCKRs were measured by the NanoBiT assay. All data are presented as mean values ± standard error of measurement (SEM). **f** Structural superposition of CCK1R (pink) and CCK2R (blue) with EP4R-G_s (purple) and GPBAR–G_s (grey). **g** CCK-8-induced cAMP accumulation and $G\alpha_q$–$G\gamma$ dissociation of $L^{5.65}$ using cAMP accumulation assay and NanoBiT assay. Bars represent differences in maximum CCK-8 response [span] for representative mutants relative to wild-type receptor (WT). NR refers to no response (or response <1% WT) when concentration of ligand changes. **h** Structural superposition of CCK2R (blue) with GPCR–G_q (grey). **i** Structural superposition of CCK1R (pink), CCK2R (blue), EP4R–G_s (purple), and GPCR–G_s (grey).

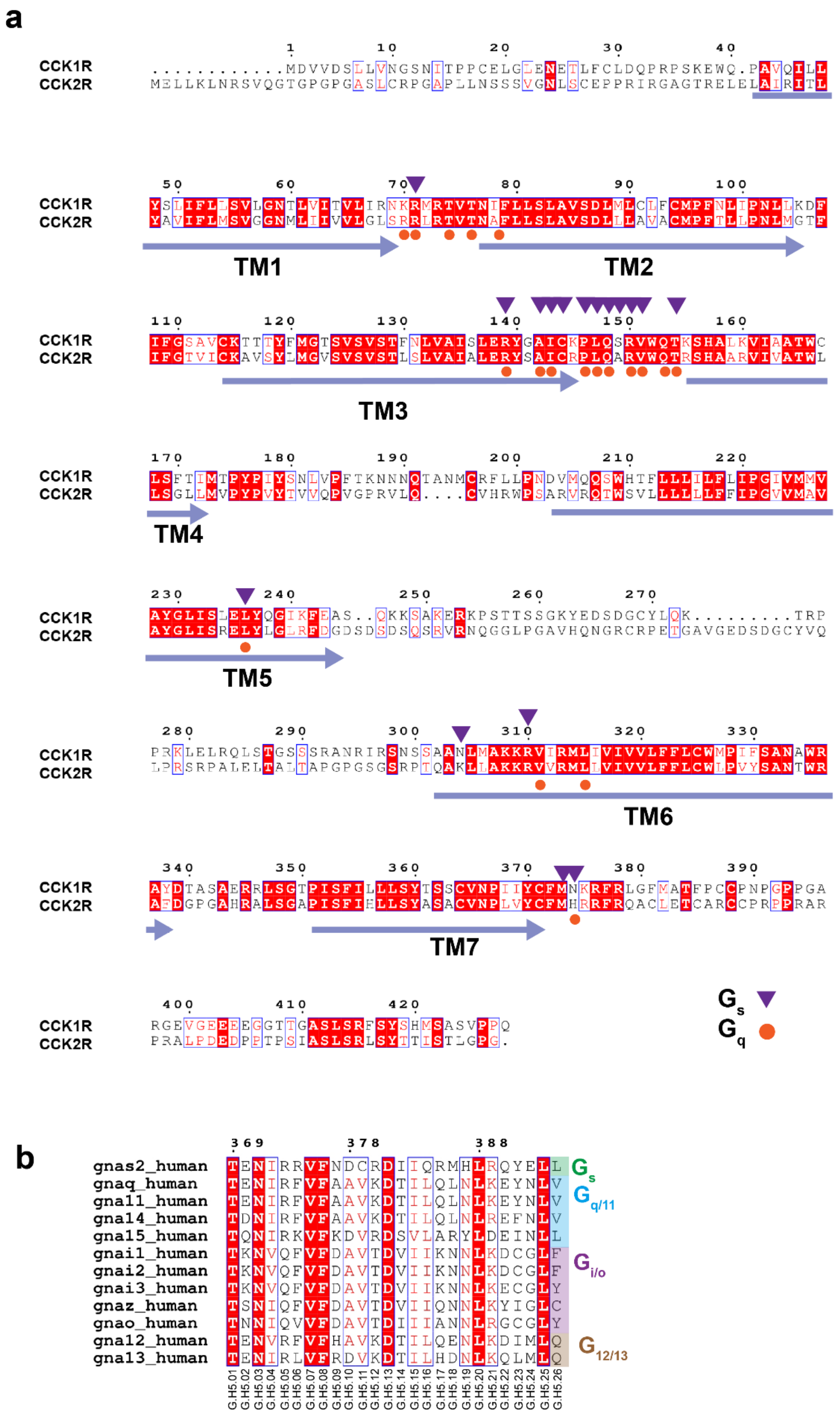

Supplementary Fig. S6 Sequence alignment of CCK1R and CCK2R and G-

protein coupling pocket differences between CCK1R and CCK2R. a Sequence alignment of CCK1R and CCK2R. Sequence alignment was performed using GPCRdb (http://www.gpcrdb. org). Different residues of G-protein coupling sites between CCK1R and CCK2R are highlighted in triangle symbols (G_s) and solid cycle (G_q). Secondary structure elements are annotated underneath sequences based on structure of CCK1R. **b** Sequence alignment of α5 of G_s, $G_{q/11}$, $G_{i/o}$, and $G_{12/13}$. Sequence alignment was performed using GPCRdb (http://www.gpcrdb. org).

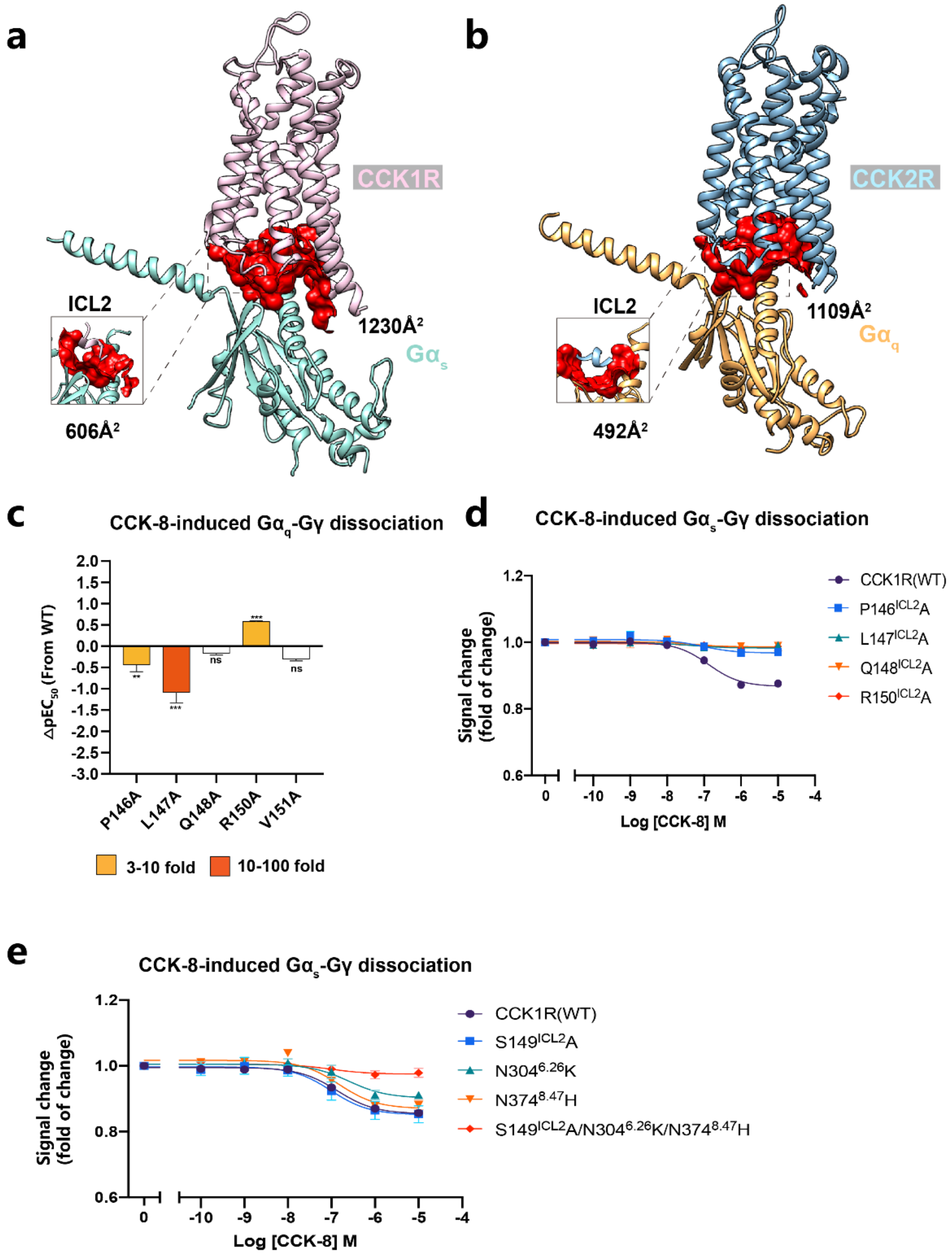

Supplementary Fig. S7 Interface between G-protein and CCK1R and CCK2R and function of ICL2 and key residues in CCK-8-induced signaling of CCK1R. a, b Interface between Gα_s and CCK1R (**a**) and interface between Gα_q and CCK2R (**b**). **c** Mutagenesis analysis of residues in ICL2 involved in Gα_s-binding site in CCK1R using NanoBiT Gα_q–Gγ dissociation assay. Bars

represent differences in calculated CCK-8 potency [pEC_{50}] for representative mutants relative to WT. Data are colored according to the extent of effect. ns, no significance, $^{**}P < 0.001$, $^{***}P < 0.0001$ (one-way ANOVA followed by Dunnett's post hoc test, compared with the response of WT). **d** Mutagenesis analysis of residues in ICL2 involved in $G\alpha_s$-binding site using NanoBiT $G\alpha_s$–$G\gamma$ dissociation assay. **e** Residues involved in G protein coupling in CCK1R are substituted by the corresponding residues in CCK2R, and the effect of each single- or triple-mutation on CCK-8–induced $G\alpha_s$–$G\gamma$ dissociation measured by NanoBiT assay is shown. All data are presented as mean values ± standard error of measurement (SEM).

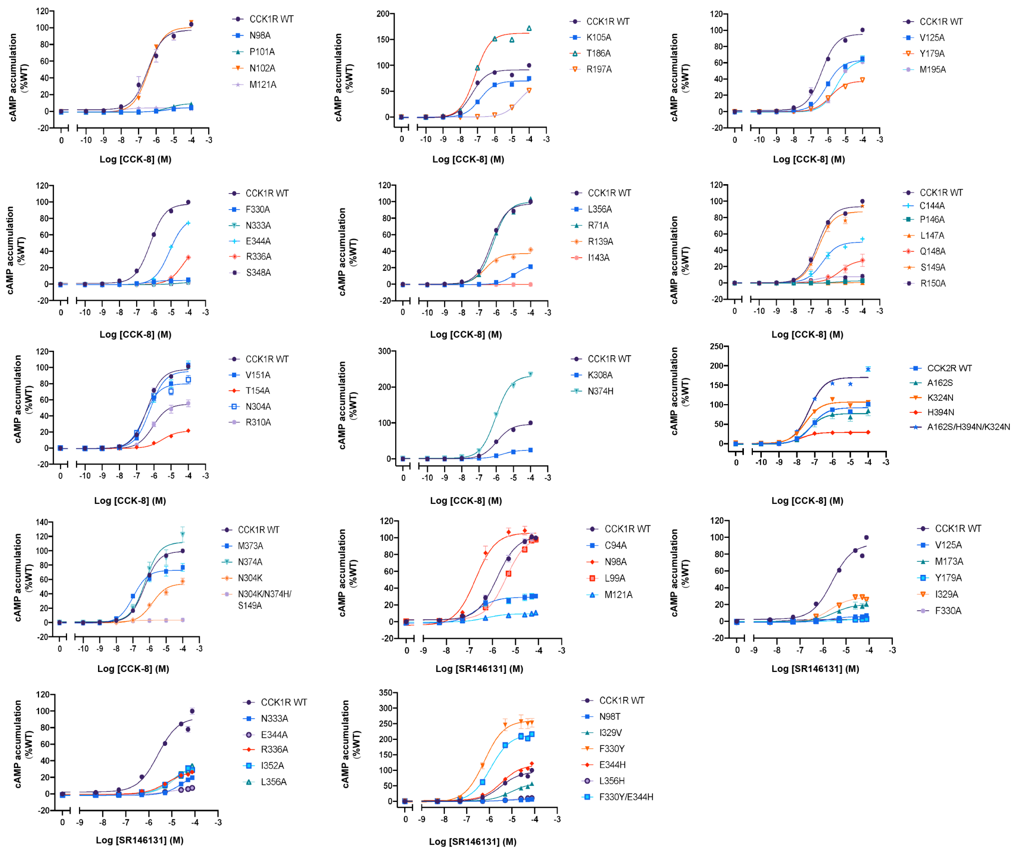

Supplementary Fig. S8 Dose response curves of ligand induced cAMP accumulation using wild type and mutant CCKRs were measured by the Glosensor assay related to Figures 2g, 4b, 4d, 6e, 7d and Tables S2, S5, S6. All data are presented as mean values ± standard error of measurement (SEM).

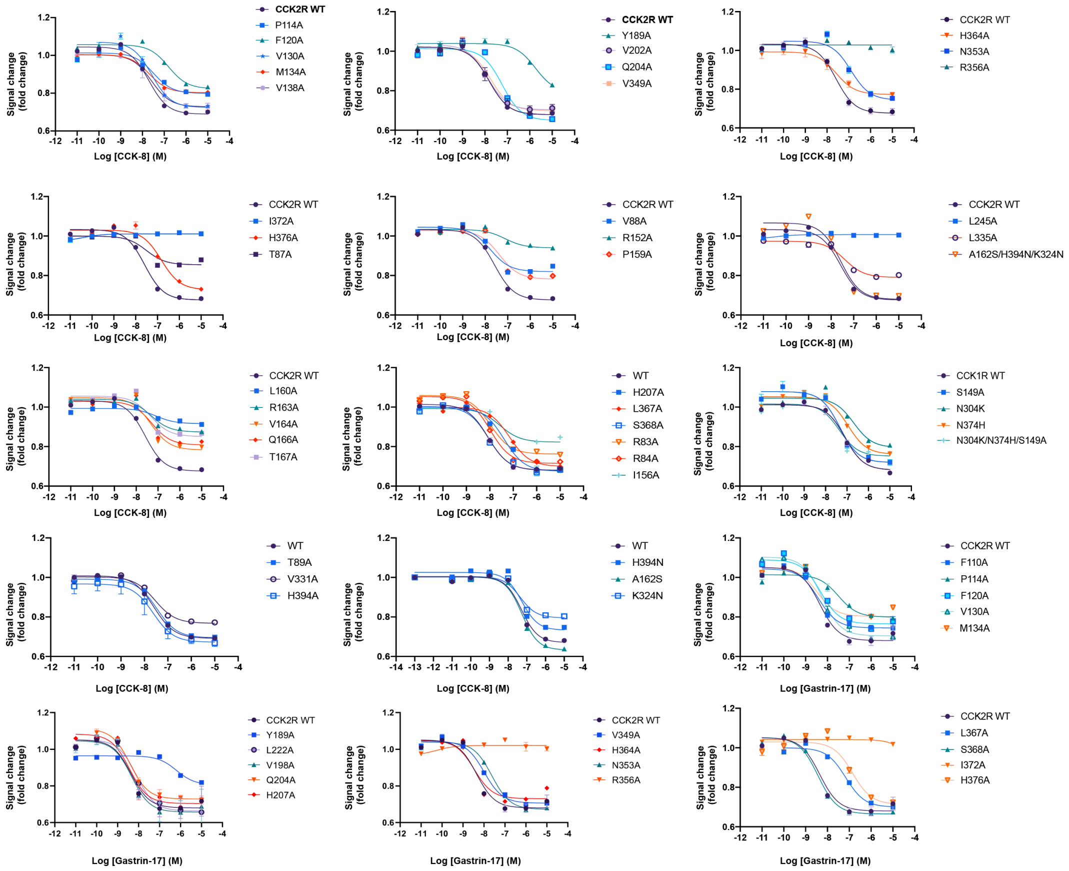

Supplementary Fig. S9 Dose response curves of ligand induced Gα_q-Gγ dissociation using wild type and mutant CCKRs were measured by the NanoBiT assay, related to Figures 2h, 2i, 6f, 7e and Tables S3, S4, S7. All data are presented as mean values ± standard error of measurement (SEM).

Table S1. Cryo-EM data collection, model refinement and validation statistics. Related to Figure 1a

	CCK-8-bound CCK1R-G_s Complex	SR146131-bound CCK1R-G_s Complex	CCK-8-bound CCK2R-G_q Complex	Gastrin-17-bound CCK2R-G_q Complex
**Data collection and processing**				
Magnification	4,9310	4,9310	4,9310	4,9310
Voltage (kV)	300	300	300	300
Electron exposure ($e^-/Å^2$)	62.4	62.4	62.4	62.4
Defocus range (μm)	-0.5 ~ -2.0	-0.5 ~ -2.0	-0.5 ~ -2.0	-0.5 ~ -2.0
Pixel size (Å)	1.014	1.014	1.014	1.014
Symmetry imposed	C1	C1	C1	C1
Initial particle projections (no.)	5,768,688	5,213,984	4,302,863	3,995,739
Final particle projections (no.)	299,092	606,684	484,441	551,048
Map resolution (Å)	3.2	3.0	3.1	3.1
FSC threshold	0.143	0.143	0.143	0.143
Map resolution range (Å)	2.2-5.0	2.2-5.0	2.2-5.0	2.2-5.0
**Refinement**				
Initial model used	7L1V, 6NBF	7L1V, 6NBF	7L1V, 6WHA	7L1V, 6WHA
Model resolution (Å)	3.2	3.0	3.1	3.1
FSC threshold	0.143	0.143	0.143	0.143
Model resolution range (Å)	2.2-5.0	2.2-5.0	2.2-5.0	2.2-5.0
Map sharpening *B* factor ($Å^2$)	-130.43	-130.13	-104.18	-142.81
Model composition				
Non-hydrogen atoms	8248	8218	8576	8566
Protein residues	1043	1036	1123	1122
B factors ($Å^2$)				
Protein	67.80	63.38	79.94	73.86
Ligand	57.68	45.41	92.95	-
R.m.s. deviations				
Bond lengths (Å)	0.003	0.003	0.003	0.004
Bond angles (°)	0.676	0.640	0.681	0.812
Validation				
MolProbity score	1.92	2.13	1.74	1.83
Clashscore	11.37	19.91	7.13	9.56
Rotamer outliers (%)	0.11	0.11	0.00	0.11
Ramachandran plot				
Favored (%)	94.91	95.19	95.00	95.26
Allowed (%)	5.09	4.81	4.90	4.74
Disallowed (%)	0.00	0.00	0.09	0.00

Table S2. CCK-8-induced cAMP accumulation in wild-type (WT) and mutant CCK1Rs. Related to Figures 2b, 5e and 7d.

	Span±SEM[a,b] (% WT)	Sample size	Expression (% WT)
WT	100±1	22	100
**Orthosteric pocket mutation**			
	**Span±SEM (% WT)**	**Sample size**	**Expression (% WT)**
N98$^{2.61}$A	3±1***	6	117±2
P101$^{2.64}$A	6±1***	3	110±5
N102$^{2.65}$A	100±6***	4	109±3
K105$^{2.68}$A	77±2	3	106±7
M121$^{3.32}$A	4±1***	5	105±3
V125$^{3.36}$A	65±2	3	78±4
Y179$^{4.63}$A	38±4 ***	4	36±3
T186^{ECL2}A	187±16***	5	95±5
M195^{ECL2}A	65±3*	4	94±7
R197^{ECL2}A	69±3	3	117±4
F330$^{6.52}$A	5±1***	3	61±5
N333$^{6.55}$A	6±2***	3	71±1
R336$^{6.58}$A	49±4***	4	106±3
E344$^{7.27}$A	74±1**	3	79±8
S348$^{7.31}$A	4±1***	3	76±4
L356$^{7.39}$A	22±2***	4	108±9
**G protein interface mutation**			
	**Span±SEM[a,b] (% WT)**	**Sample size**	**Expression (% WT)**
R71^{ICL1}A	103±4	4	115±7
R139$^{3.50}$A	42±4**	4	32±5
I143$^{3.54}$A	NR	4	5±0
C144$^{3.55}$A	50±5*	3	60±3
P146^{ICL2}A	NR	4	51±3
L147^{ICL2}A	NR	4	111±6
Q148^{ICL2}A	28±5***	3	114±5
S149^{ICL2}A	89±3	3	97±3
R150^{ICL2}A	8±1***	5	98±4
V151^{ICL2}A	94±6	4	116±5
T154$^{4.38}$A	22±3***	3	83±3
N304$^{6.26}$A	88±11	3	116±2
K308$^{6.30}$A	25±1***	3	99±1
R310$^{6.32}$A	52±6**	5	117±6
M373$^{7.56}$A	76±8	3	67±6

N374$^{8.47}$A	113±16	4	101±13
**G protein interface corresponding mutation**			
N304$^{6.26}$K	56±10**	3	61±1
N374$^{8.47}$H	240±7***	4	81±2
N304$^{6.26}$K/N374$^{8.47}$H/S149^{ICL2}A	3±0***	4	39±2

[a]Data shown are means ± SEM from at least three independent experiments performed in technical triplicate. *P<0.01; **P<0.001 and ***P<0.0001 by one-way ANOVA followed by Dunnett's post-test, compared with the response of the WT.

[b]The span is defined as the window between the maximal response (E_{max}) and the vehicle (no CCK-8). NR (no response) refers to no response (or response < 1% WT) occurred as the concentration of ligand changes.

Table S3. CCK-8-induced Gα_q-Gγ dissociation in wild-type (WT) and mutant CCK2Rs. Related to Figures 2d, 5f and 7e.

	**Span±SEM[a,b] (% WT)**	**Sample size**	**Expression (% WT)**
WT	100±1	20	100
**Orthosteric pocket mutation**			
	**Span±SEM (% WT)**	**Sample size**	**Expression (% WT)**
P114$^{2.64}$A	57±6***	3	58±3
F120^{ECL1}A	65±4***	4	97±10
V130$^{3.28}$A	102±2	3	110±3
M134$^{3.32}$A	60±4***	5	97±5
V138$^{3.36}$A	87±3	4	58±6
Y189$^{4.60}$A	73±2*	3	55±3
V202^{ECL2}A	99±1	3	106±0
Q204^{ECL2}A	100±0	3	97±3
H207^{ECL2}A	110±10	3	110±2
V349$^{6.51}$A	97±7	4	100±1
N353$^{6.55}$A	88±4	4	107±6
R356$^{6.58}$A	NR	4	42±3
H364$^{7.27}$A	NR	3	54±2
L367$^{7.30}$A	96±3	3	90±4
S368$^{7.31}$A	97±4	4	117±9
I372$^{7.35}$A	NR	4	16±1
H376$^{7.39}$A	82±3	3	93±5
**G protein interface mutation**			
	**Span±SEM[a,b] (% WT)**	**Sample size**	**Expression (% WT)**
R83^{ICL1}A	88±3	4	62±2
R84^{ICL1}A	91±5	4	73±11
T87$^{2.37}$A	33±4***	4	63±4
V88$^{2.38}$A	59±2***	5	110±8
T89$^{2.39}$A	95±8	3	105±9
R152$^{3.50}$A	30±2***	5	108±6
I156$^{3.54}$A	43±4***	3	61±3
P159^{ICL2}A	68±7***	3	73±0
L160^{ICL2}A	27±3***	3	122±8
R163^{ICL2}A	45±3***	4	69±2
V164^{ICL2}A	69±6***	3	85±2
Q166^{ICL2}A	57±4***	4	114±4
T167$^{4.38}$A	52±2***	6	93±4
L245$^{5.65}$A	NR	4	7±1

$V331^{6.33}A$	81±3	3	81±7
$L335^{6.37}A$	52±2***	4	65±5
$H394^{8.47}A$	100±6	3	71±1
**G protein interface corresponding mutation**			
$A162^{ICL2}S$	116±3	3	101±2
$K324^{6.26}N$	70±5	3	44±7
$H394^{8.47}N$	91±2	3	56±3
$A162^{ICL2}S/H394^{8.47}N/K324^{6.26}N$	102±2	3	60±2

[a]Data shown are means ± SEM from at least three independent experiments performed in technical triplicate. *P<0.01; **P<0.001 and ***P<0.0001 by one-way ANOVA followed by Dunnett's post-test, compared with the response of the WT.

[b]The span is defined as the window between the maximal response (E_{max}) and the vehicle (no CCK-8). NR (no response) refers to no response (or response < 1% WT) occurred as the concentration of ligand changes.

Table S4. Gastrin-17-induced Gα_q-Gγ dissociation in wild-type (WT) and mutant CCK2Rs. Related to Figures 2e.

	**Span±SEM[a,b] (% WT)**	**Sample size**	**Expression (% WT)**
WT	100±1	20	100
**Orthosteric pocket mutation**			
	**Span±SEM (% WT)**	**Sample size**	**Expression (% WT)**
F110$^{2.60}$A	81±2	5	68±4
P114$^{2.64}$A	67±2*	5	67±5
F120^{ECL1}A	90±4	3	97±10
V130$^{3.28}$A	110±7	4	110±3
M134$^{3.32}$A	67±3	3	97±5
Y189$^{4.60}$A	47±4***	4	71±1
V198^{ECL2}A	106±6	3	124±13
Q204^{ECL2}A	102±6	4	98±3
H207^{ECL2}A	104±7	4	165±6
L222$^{5.42}$A	111±4	3	125±3
V349$^{6.51}$A	92±4	3	100±1
N353$^{6.55}$A	95±2	4	113±9
R356$^{6.58}$A	NR	3	36±4
H364$^{7.27}$A	53±9***	4	59±2
L367$^{7.30}$A	81±1	4	91±4
S368$^{7.31}$A	105±7	4	117±9
I372$^{7.35}$A	NR	3	18±1
H376$^{7.39}$A	72±4	3	93±5

[a]Data shown are means ± SEM from at least three independent experiments performed in technical triplicate. *P<0.01; **P<0.001 and ***P<0.0001 by one-way ANOVA followed by Dunnett's post-test, compared with the response of the WT.

[b]The span is defined as the window between the maximal response (E_{max}) and the vehicle (no gastrin-17). NR (no response) refers to no response (or response < 1% WT) occurred as the concentration of ligand changes.

Table S5. SR146131-induced cAMP accumulation in wild-type (WT) and mutant CCK1Rs. Related to Figures 4b and 4d.

	**Span±SEM[a,b] (% WT)**	**Sample size**	**Expression (% WT)**
WT	100±1	18	100
**Orthosteric pocket mutation**			
	**Span±SEM (% WT)**	**Sample size**	**Expression (% WT)**
$C94^{2.57}A$	33±2**	4	123±3
$N98^{2.61}A$	113±6	3	117±2
$L99^{2.62}A$	96±7	4	113± 3
$M121^{3.32}A$	8±0***	5	105±3
$V125^{3.36}A$	13±1***	5	82±4
$M173^{4.57}A$	23±4***	3	124±12
$Y179^{4.63}A$	3±0***	5	33±1
$I329^{6.51}A$	30±3**	3	76±8
$F330^{6.52}A$	NR	3	67±2
$N333^{6.55}A$	22±2***	5	71±1
$R336^{6.58}A$	21±3***	3	106±3
$E344^{7.27}A$	7±1***	4	79±8
$I352^{7.35}A$	37±1***	3	108±1
$L356^{7.39}A$	40±5***	3	97±6
**Orthosteric pocket corresponding mutation**			
	**Span±SEM[a,b] (% WT)**	**Sample size**	**Expression (% WT)**
$N98^{2.61}T$	8±1***	4	66±3
$I329^{6.51}V$	67±6	4	106±2
$F330^{6.52}Y$	274±25***	3	106±4
$E344^{7.27}H$	101±4	3	99±2
$L356^{7.39}H$	12±2***	3	105±5
$F330^{6.52}Y/E344^{7.27}H$	248±24***	3	100±3

[a]Data shown are means ± SEM from at least three independent experiments performed in technical triplicate. *P<0.01; **P<0.001 and ***P<0.0001 by one-way ANOVA followed by Dunnett's post-test, compared with the response of the WT.

[b]The span is defined as the window between the maximal response (E_{max}) and the vehicle (no SR146131). NR (no response) refers to no response (or response < 1% WT) occurred as the concentration of ligand changes.

Table S6. CCK-8-induced cAMP accumulation in wild-type (WT) and mutant CCK2Rs. Related to Figures 7d.

	**Span±SEM[a,b] (% WT)**	**Sample size**	**Expression (% WT)**
WT	100±1	6	100
**Orthosteric pocket mutation**			
	**Span±SEM (% WT)**	**Sample size**	**Expression (% WT)**
$A162^{ICL2}S$	83±11	4	93±6
$K324^{6.26}N$	98±8	4	47±5
$H394^{8.47}N$	27±1	4	71±1
$A162^{ICL2}S/H394^{8.47}N/K324^{6.26}N$	190±5***	3	62±2

[a]Data shown are means ± SEM from at least three independent experiments performed in technical triplicate. *P<0.01; **P<0.001 and ***P<0.0001 by one-way ANOVA followed by Dunnett's post-test, compared with the response of the WT.

[b]The span is defined as the window between the maximal response (E_{max}) and the vehicle (no CCK-8). NR (no response) refers to no response (or response < 1% WT) occurred as the concentration of ligand changes.

Table S7. CCK-8-induced $G\alpha_q$-$G\gamma$ dissociation in wild-type (WT) and mutant CCK1Rs. Related to Figures 7E.

	**Span±SEM[a,b] (% WT)**	**Sample size**	**Expression (% WT)**
WT	100±1	6	100
**Orthosteric pocket mutation**			
	**Span±SEM (% WT)**	**Sample size**	**Expression (% WT)**
N304$^{6.26}$K	78±2	3	61±1
N374$^{8.47}$H	91±1	3	81±1
S149A	89±3	3	97±3
N304$^{6.26}$K/N374$^{8.47}$H/S149^{ICL2}A	79±1	5	37±3

[a]Data shown are means ± SEM from at least three independent experiments performed in technical triplicate. *P<0.01; **P<0.001 and ***P<0.0001 by one-way ANOVA followed by Dunnett's post-test, compared with the response of the WT.

[b]The span is defined as the window between the maximal response (E_{max}) and the vehicle (no CCK-8). NR (no response) refers to no response (or response < 1% WT) occurred as the concentration of ligand changes.
